# A STIM2 splice variant negatively regulates store-operated calcium entry

**DOI:** 10.1038/ncomms7899

**Published:** 2015-04-21

**Authors:** Anna-Maria Miederer, Dalia Alansary, Gertrud Schwär, Po-Hsien Lee, Martin Jung, Volkhard Helms, Barbara A. Niemeyer

**Affiliations:** 1 Molecular Biophysics, Center for Integrative Physiology and Molecular Medicine (CIPMM), School of Medicine, Saarland University, Building 48, Homburg 66421, Germany; 2 Department of Biophysics, Center for Integrative Physiology and Molecular Medicine (CIPMM), School of Medicine, Saarland University, Homburg 66421, Germany; 3 Center for Bioinformatics, Saarland University, Campus E2 1, R. 315, PO Box 151150, Saarbrücken 66041, Germany; 4 Department of Medical Biochemistry and Molecular Biology, Saarland University, Building 44, Homburg 66421, Germany

## Abstract

Cellular homeostasis relies upon precise regulation of Ca^2+^ concentration. Stromal interaction molecule (STIM) proteins regulate store-operated calcium entry (SOCE) by sensing Ca^2+^ concentration in the ER and forming oligomers to trigger Ca^2+^ entry through plasma membrane-localized Orai1 channels. Here we characterize a *STIM2* splice variant, *STIM2.1*, which retains an additional exon within the region encoding the channel-activating domain. Expression of *STIM2.1* is ubiquitous but its abundance relative to the more common *STIM2.2* variant is dependent upon cell type and highest in naive T cells. STIM2.1 knockdown increases SOCE in naive CD4^+^ T cells, whereas knockdown of STIM2.2 decreases SOCE. Conversely, overexpression of STIM2.1, but not STIM2.2, decreases SOCE, indicating its inhibitory role. STIM2.1 interaction with Orai1 is impaired and prevents Orai1 activation, but STIM2.1 shows increased affinity towards calmodulin. Our results imply STIM2.1 as an additional player tuning Orai1 activation *in vivo*.

## Abstract (web summary)

STIM proteins sense calcium depletion in the endoplasmic reticulum and in response activate calcium influx through Orai1 channels located at the plasma membrane. Here, Miederer *et al*. identify a novel splice variant of STIM2 that fails to interact with and activate Orai1 and may act to fine-tune cellular calcium homeostasis by negatively regulating calcium influx.

## Graphical abstract

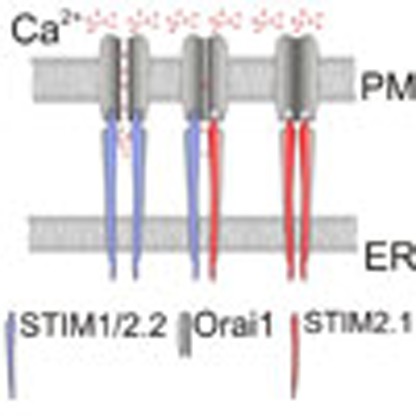

Amplitude, duration and kinetics of Ca^2+^ signals code for differential changes in gene expression, degranulation, cytokine release, proliferation and migration. These diverse functions require tight control and regulation of Ca^2+^ influx. In immune cells, the major influx pathway for Ca^2+^ is encoded by components of the store-operated calcium entry (SOCE) machinery. Here, CRAC (Ca^2+^ release-activated Ca^2+^) channels encoded by the Orai genes (Orai1-3) are activated by endoplasmatic reticulum (ER)-localized stromal interaction molecule (STIM) (STIM1 and STIM2) proteins when the Ca^2+^ concentration of the ER (∼400–600 μM^1^) is decreased^2^. In contrast to STIM1, the major activator of Orai1 upon effector T-cell activation, the functional role of STIM2 is less well understood. The first studies on STIM2 in 2006 described an inhibitory effect of STIM2 on STIM1-mediated SOCE, as well as its constitutive and store-independent activation of Orai1 (ref. 3). An short interfering RNA (siRNA)-based screen for regulators of basal cytosolic Ca^2+^ identified STIM2, calmodulin (CaM) and the plasma membrane Ca^2+^ ATPase (PMCA) as critical components with downregulation of STIM2 decreasing basal Ca^2+^ and downregulation of CaM or plasma membrane Ca^2+^ ATPase increasing basal [Ca^2+^] (ref. 4). The authors also showed that STIM2 responds to smaller decreases in ER [Ca^2+^] with an EC50 of 406 μM compared with 210 μM for STIM1 (see also ref. 5). In addition to the decreased affinity of the Ca^2+^-binding EF hand, the STIM2 SAM domain displays an increased conformational stability when compared with STIM1 (ref. 5). In contrast to STIM1, the SAM domain of STIM2 contains only a single *N*-glycosylation site. Modifications of the glycosylation sites within the SAM domain of STIM1 lead to altered oligomerization rates and current size as well described by a reaction diffusion model^6^.

Whether the effect of STIM2 on SOCE is activation or inhibition is controversial, and a recent published computational model of regulation of basal Ca^2+^ homeostasis did not consider a role of STIM2 (ref. 7). A second confounding factor in the analysis of STIM2 function is an existing uncertainty regarding its translational start site. In the original report describing cloning of STIM2, Williams *et al*.^8^ postulated translation from a non-AUG codon around L88, a view that was later revised by Graham *et al*., who showed that STIM2 contains an unconventionally long signal peptide with translation from a conserved Met, which can cause incomplete protein translocation into the ER and leads to a small amount of cytosolic STIM2, responsible for pre-coupled and non-store-operated activation of STIM2–Orai1 complexes^9^. In addition to its role in maintaining basal Ca^2+^and controlling ER Ca^2+^ levels^4^, STIM2 activates Orai1 signalling upon submaximal store depletion, driving Ca^2+^ oscillations due to partially pre-coupled STIM2–Orai1 population^10^,^11^,^12^,^13^,^14^. STIM2 and Orai1 form a Ca^2+^-sensitive and thapsigargin-insensitive complex in cortical neurons^15^, with loss of STIM2-protecting neurons from store-mediated hypoxic neuronal death^16^. In cancer, controversial roles for STIM2 as both a tumour suppressor or as a potential oncogene have been postulated (17,18,19,20, reviewed in ref. 14). Recent publications also present growing evidence for a key role for STIM2 in tumour immunity by CD8^+^ cells^21^ and in several immune diseases. In a murine multiple sclerosis model, STIM2-deficient mice were shown to be less susceptible to autoimmune encephalomyelitis (EAE), suggesting a role for STIM2-mediated Ca^2+^ signalling in autoimmune disease^22^,^23^. In contrast to the EAE model, deficient STIM2 signalling caused salivary gland autoimmune pathology in Primary Sjögren's Syndrome^24^.

Here we report the identification and detailed characterization of a novel *STIM2* splice variant, named *STIM2.1*, which acts as an inhibitory regulator of STIM-mediated activation of Orai channels. The additional exon of *STIM2.1* is spliced into the channel-activating domain (CAD), immediately upstream of the sequences essential for binding of Orai1 (refs 13, 25), disabling STIM2.1 CAD from activating Orai1 and altering the CAD domain affinity for CaM binding.

## Results

### Identification of a novel *STIM2* splice variant

**Figure 1:**
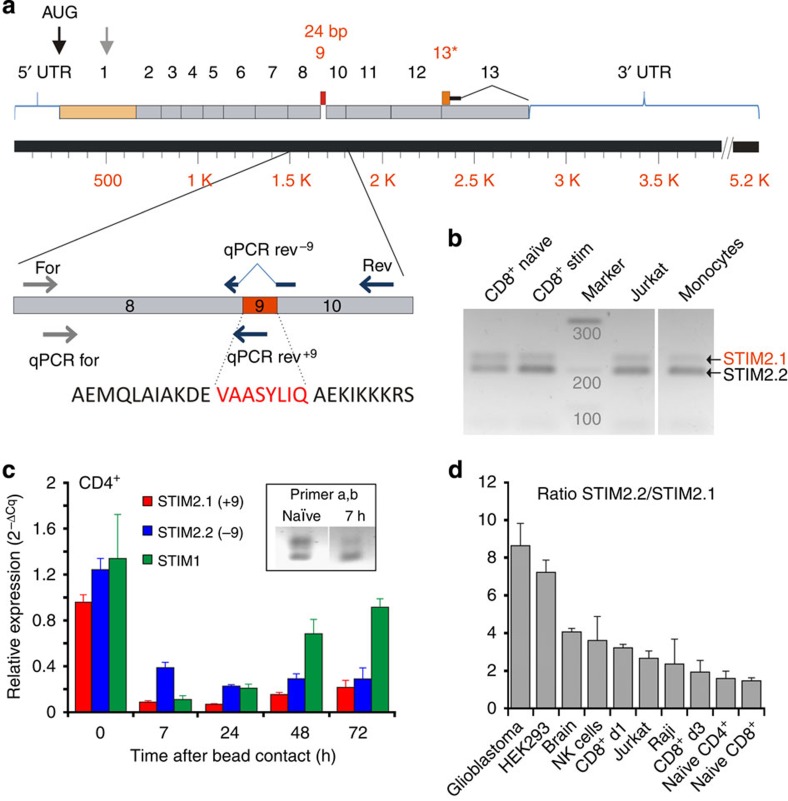
Identification of a novel STIM2 splice variant. (**a**) Schematic representation of human STIM2 mRNA with exon boundaries. Highlighted in red are exon 9 and 13* present only in the splice variants *STIM2.1* and *STIM2*.*3*, respectively. The enlarged region shows the primer pairs used in conventional (for: grey arrow and rev: blue arrow) or quantitative (qPCR for, qPCR rev −9, qPCR rev +9) PCRs to detect and analyse splice variant expression. (**b**) Image showing PCR amplification products obtained with primer pair (for and rev) using cDNA from naive and stimulated human CD8^+^T cells, Jurkat T cells and human monocytes. (**c**) Relative expression of *STIM1*, *STIM2.1* and *STIM2.2* in naive and stimulated CD4^+^ T cells with indicated time periods after initial contact with anti-CD3/anti-CD28-coated beads. Expression was normalized to that of TBP (three donors) (**d**) Ratio of *STIM2.2*/*STIM2.1* expression obtained by qRT–PCR using reverse-transcribed mRNA isolated from the cell types indicated below the bars (3–12 donors or independent RNA preparations).

We discovered the existence of two additional *STIM2* splice variants by database mining, namely *STIM2.1* containing an additional exon 9 and *STIM2.3* containing an alternative exon 13 (13*) leading to an upstream end of translation and a transcript shortened by 444 bp (∼17 kDa) (Fig. 1a). All reports on STIM2 are conducted with the STIM2.2 variant. Although we were unable to detect messenger RNA (mRNA) expression of *STIM2.3* in lymphocytes by different PCR-based strategies, we identified *STIM2.1* in a conventional PCR reaction with primers (for, rev) flanking exon 9. Figure 1b shows two different PCR products in human CD8^+^ T cells as well as in Jurkat T cells and primary monocytes. Exon 9-specific quantitative reverse transcription (RT)–PCR primers were derived (Fig. 1a; Supplementary Table 1), PCR products were confirmed by DNA sequencing and relative expression levels of *STIM2.1* (with exon 9, NM001169118) and of *STIM2.2* (without exon 9, NM020860) were tested using template complementary DNA (cDNA) of naive and stimulated CD4^+^ T cells from at least three different primary human blood donors. As also indicated by conventional PCR (inset in Fig. 1c), *STIM2.1* expression is highest in naive T cells but is reduced upon stimulation with anti-CD3/anti-CD28-coated beads. Seven hours after bead contact, the ratio of *STIM2.2*/*STIM2.1* transiently increases to 4±0.5, but decreases again to a ratio of 1.6±0.56 after 72 h following initial bead contact (Fig. 1c). A reduction of mRNA expression is also seen for *STIM2.2* and for *STIM1* although *STIM1* expression recovers after 72 h, whereas total *STIM2* mRNA and STIM2 protein remains reduced in stimulated cells (Fig. 1c; Supplementary Fig. 1a,b). We proceeded to test splice-specific expression in a number of cell lines and tissues and plotted the ratio of *STIM2.2* expression over *STIM2.1* expression (Fig. 1d). Highest expression of the novel *STIM2.1* (lowest ratio) is detected in naive CD4^+^ and CD8^+^ T cells. cDNA from glioblastoma samples (12 patients) showed the highest expression of *STIM2.2* with little *STIM2.1*. In summary, we could detect expression of *STIM2.1* in all tested human cell lines and primary cells, although expression was always lower than *STIM2.2*. Alignment of the exon 9 amino acids (VASSYLIQ) shows a high degree of conservation between different species. We also tested for *STIM2.1* (VAASYLLQ) expression in lymphocytes from *Mus musculus* and were able to detect two bands by conventional PCR (Supplementary Fig. 1d).

### Knockdown of *STIM2.1* alters SOCE in primary cells

**Figure 2:**
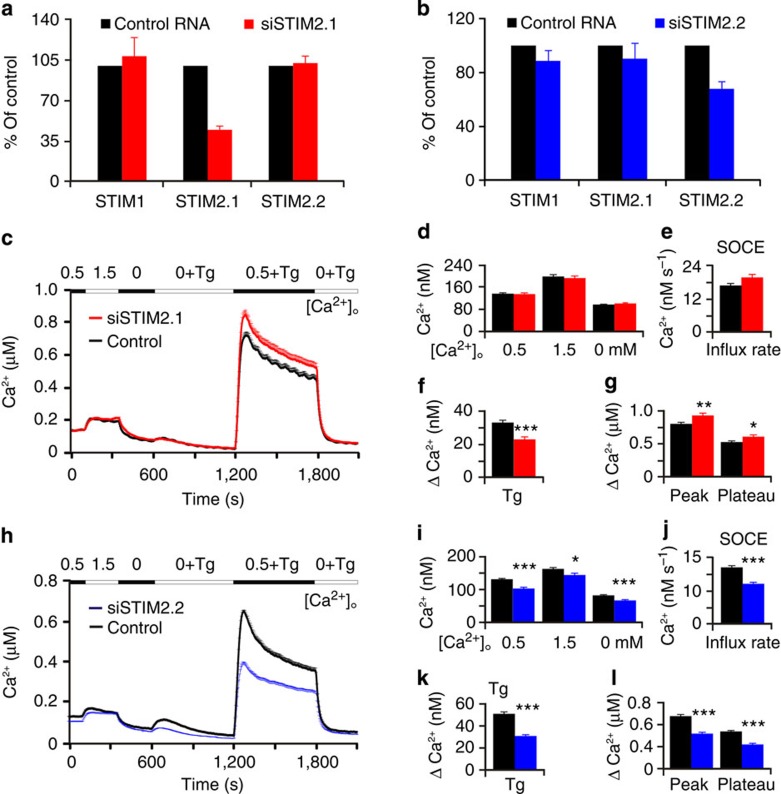
Specific knockdown of *STIM2.1* alters SOCE in primary cells. (**a**) Relative expression of *STIM1*, *STIM2.1* and *STIM2.2* after transfection with siRNA against exon 9 (*STIM2.1*) red or siRNA against exon 8/10 boundary (**b**, *STIM2.2*, blue), corresponding control transfection, black bars. (**c**) Traces showing average changes in intracellular Ca^2+^ concentration [Ca^2+^]_i_ over time in CD4^+^ T cells transfected with control (black, *n*=698) or siRNA against *STIM2.1* (red, *n*=579) in response to perfusion of different [Ca^2+^]_o_ indicated in the upper bar. (**d**–**g**) Quantification of changes in [Ca^2+^]_i_ measured in **c**. (**h**) Traces showing changes in [Ca^2+^]_i_ in CD4^+^ T cells treated as in **c** after transfection with control (black, *n*=978) or siRNA against *STIM2.2* (blue, *n*=776). (**i**–**l**) Quantification of changes in [Ca^2+^]_i_ measured in h. Measurements shown in **c**–**l** are obtained from cells of three independent blood donors and ⩾8 experiments. *P<0.05, **P<0.01, ***P<0.001, Student's *T*-test. Data obtained are presented as mean±s.e.m.

Because naive CD4^+^ and CD8^+^ T cells showed the highest absolute expression of *STIM2.1* (2^−ΔCq^: 0.68±0.35), with an average ratio of *STIM2.2*/*STIM2.1* expression of 1.6±0.4 (5 donors) for naive CD4^+^ cells and a ratio of 1.5±0.14 (3 donors) for CD8^+^ cells (see Fig. 1c), these cells lent themselves for investigating endogenous STIM2.1 function. Although limited by the very short sequence of exon 9, we devised splice-specific siRNA targeting either exon 9 or the exon 8/exon 10 boundary (Supplementary Table 1). Efficiency of knockdown was tested by qRT–PCR 14–18 h after siRNA transfections on two consecutive days. *STIM2.1*-specific siRNA decreased cDNA expression to an average of 44±6% of its non-silenced control without exerting a significant effect on the expression of *STIM2.2* or on *STIM1* (Fig. 2a), neither did the siRNA show off-target or indirect effects on the expression of Orai1 (97% of control). Given an mRNA expression ratio of *STIM2.2*/*STIM2.1* of 1.6 and a knockdown efficiency of ∼50%, we expected a reduction of total STIM2 protein of about 20%, which we indeed observed (Supplementary Fig. 1c). Splice-specific knockdown of *STIM2.2* was not as successful, leading to reduction of *STIM2.2* expression to an average of 68±8% and to small (∼10%) off-target or indirect effects on *STIM1*, *STIM2.1* (Fig. 2b) and Orai1 (91% of control) expression. Measurements of [Ca^2+^]_i_ of naive T cells showed that siRNA specific to *STIM2.1* did not have a significant effect on basal [Ca^2+^]_i_; however, it led to a significant increase in rate, peak and plateau of SOCE (Fig. 2c–g). In contrast and despite the relatively weak downregulation of *STIM2.2* expression, Ca^2+^ imaging of primary naive CD4^+^ cells revealed that *STIM2.2* siRNA significantly decreased basal Ca^2+^ concentrations in bath solutions with different [Ca^2+^]_o_, (Fig. 2h,i), as expected upon downregulation of STIM2 (ref. 4), decreased the size of the thapsigargin (Tg)-releasable pool, which is very small and variable in naive T cells (see Supplementary Fig. 1e) and significantly reduced SOCE (Fig. 2j–l), demonstrating that expression of STIM2.2 in naive cells is a significant determinant of their Ca^2+^ homeostasis, and confirming that STIM2.2 is a regulator not only of basal but also of store-operated Ca^2+^ homeostasis. *STIM2.1* siRNA treatment showed opposite effects, strongly indicating that STIM2.1 acts as a break or inhibitory regulator on STIM1/STIM2-mediated SOCE *in vivo*.

### Upregulation of STIM2.1 decreases T-cell SOCE

**Figure 3:**
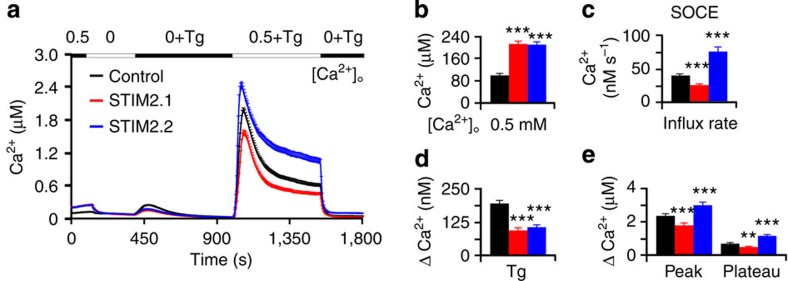
Upregulation of STIM2.1 decreases T-cell SOCE. (**a**) Traces showing average changes in intracellular Ca^2+^ concentration [Ca^2+^]_i_ over time in Jurkat T cells transfected with control (black), *STIM2.1*-mCherry (red) or *STIM2.2*-mCherry (blue) in response to perfusion of different [Ca^2+^]_o_ indicated in the upper bar. (**b**–**e**) Quantification of changes in [Ca^2+^]_i_ measured in **a** from 131 to 216 cells of 6 experiments. **P<0.01, ***P<0.001, Student's *T*-test. Data obtained are presented as mean±s.e.m.

If downregulation of STIM2.1 increases SOCE, we reasoned that upregulation of STIM2.1 in Jurkat T cells may decrease SOCE and therefore expressed either *STIM2.2* or *STIM2.1* after cloning exon 9 into a STIM2 expression vector with the full STIM2 signal peptide sequence and where the fluorescent mcherry-coding sequence replaced the variable domain NT 1966–2160 (I648-K711), retaining 121 C-terminal aa residues (see also below). Figure 3a shows that overexpression of STIM2.2 in Jurkat T cells leads to an increased basal [Ca^2+^], a reduced Tg-releasable store content but also an increased rate, peak and plateau of SOCE (Fig. 3b–e). Overexpression of STIM2.1 showed similar effects to STIM2.2 on basal Ca^2+^ and Tg-releasable store content but, in contrast to STIM2.2, significantly decreased rate, peak and plateau of SOCE (Fig. 3c,e). These results complement the siRNA data concerning SOCE (Fig. 2) and confirm an opposite role for both splice variants in regulation of SOCE after full store depletion.

### STIM2.1 is unable to activate Orai1

**Figure 4:**
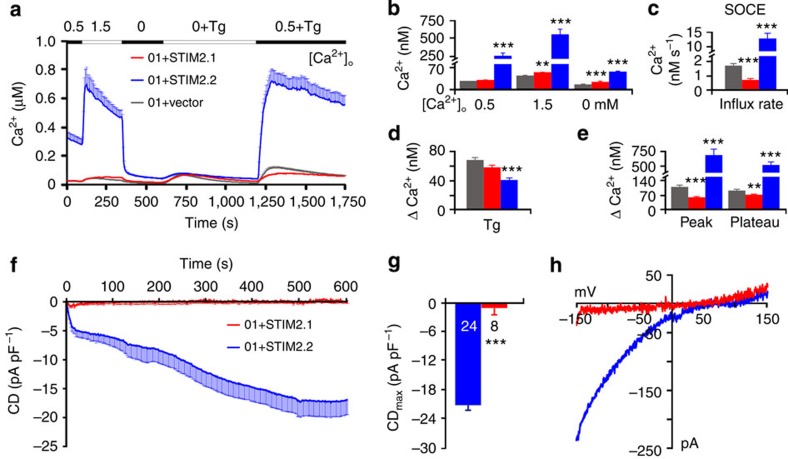
STIM2.1 is unable to activate Orai1 in overexpression systems. (**a**) Traces showing average changes in intracellular Ca^2+^ concentration [Ca^2+^]_i_ over time in response to perfusion of different [Ca^2+^]_o_ indicated in the upper bar in HEKO1 cells transfected with YFP-*STIM2.1* (red, *n*=96) or YFP-*STIM2.2* (blue, *n*=107) or an empty vector control (grey, *n*=72), 6 experiments per condition. (**b**–**e**) Quantification of changes in [Ca^2+^]_i_ measured in **a**. (**f**) Average traces showing whole-cell current density (CD) over time extracted at −130 mV in HEKO1 cells transfected with YFP-*STIM2.1* (red) or YFP-*STIM2.2* (blue). (**g**) Average maximum CD recorded from cells measured in **f**. (**h**) Current–voltage (*I*–*V*) relationship of representative cells recorded in **f**. **P<0.01, ***P<0.001, Student's *T*-test. Data obtained are presented as mean±s.e.m.

To further analyse the function of STIM2.1, we cloned exon 9 into an existing *STIM2.2* expression vector, where the STIM1 signal peptide sequence is followed by YFP and the sequence of STIM2 starting at C102 (ref. 4) or into a vector containing the full STIM2 signal peptide (M1-G101) and coding sequence with no added fluorescent tag^10^. Expression of each of these constructs in HEK293 cells stably expressing Orai1 (HEKO1) cells revealed a significant increase in the basal Ca^2+^ content of the cytosol upon expression of STIM2.2, as well as a significant increase in SOCE when compared with an equimolar vector control. Figure 4a–e shows the results for the N-terminally tagged constructs (see also ref. 4). Expression of *STIM2.1* alone did not increase basal [Ca^2+^]_i_ nor significantly affected the Tg-releasable pool. However, in contrast to STIM2.2, STIM2.1 is not only unable to activate SOCE upon store depletion but also acts as an inhibitor of SOCE in the vector-only-transfected control (Fig. 4a–e). The YFP or mcherry fluorescence of the directly tagged constructs (Fig. 3) clearly indicated expression, which was also confirmed by western blot analysis (Supplementary Fig. 1f). To directly measure channel activity, we conducted whole-cell patch-clamp recordings of STIM2.1 and STIM2.2 expressed in HEKO1 cells with a pipette solution containing IP_3_ and BAPTA (1,2-bis(o-aminophenoxy)ethane-*N,N,N′,N′*-tetraacetic acid). As expected, STIM2.2 displayed inward currents that showed an initial rapid phase (store operated) and a second slower phase of increasing current density indicative of loss of a diffusive factor from the cytosol^10^ (Fig. 4f). Similar to the results shown in Fig. 4a, STIM2.1 was unable to sustain Orai1 currents (Fig. 4f–h). We also tested the ability of STIM2.1 and STIM2.2 to activate Orai2 channels and obtained a related result, namely a large increase in basal Ca^2+^ and Orai2-mediated SOCE can be observed with STIM2.2 but not with STIM2.1 (Supplementary Fig. 1g–k). The results shown in Fig. 4 indicate that STIM2.1 by itself either may be unable to cluster and localize to ER–PM (plasma membrane) junctional regions, unable to bind to and/or unable to gate Orai1 channels effectively. Thus, we set out to investigate the mechanisms underlying the observed functional differences between STIM2.1 and STIM2.2.

### STIM2.1 interaction with Orai1 is impaired

**Figure 5:**
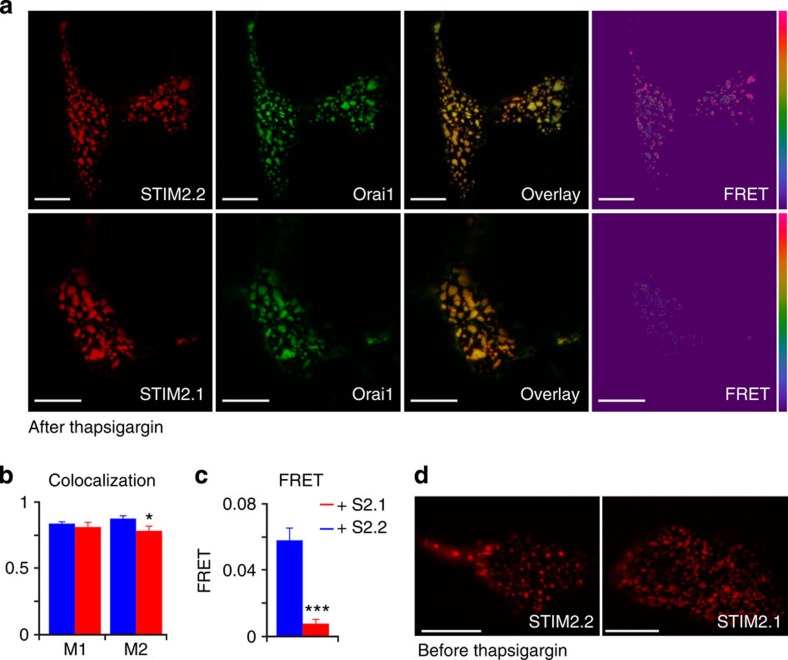
Co-localization and interaction analyses. (**a**) Images showing HEKWT cells expressing Orai1-GFP (green), STIM2.1- (upper panel) or STIM2.2 (lower panel)-mCherry (red) and the corresponding overlay and FRET images (LUT 0–0.3). (**b**) Analysis of co-localization of STIM2 and Orai1 using Manders coefficient (M1) or vice versa (M2) (*n*=9 images for both conditions) in the same cells as analysed for FRET (STIM2.1-Orai1 *n*=36; STIM2.2-Orai1 *n*=31, from 3 independent experiments). (**c**) Quantification of the FRET signals measured in **a**. *P<0.05, ***P<0.001, Student *T*-test. Data obtained are presented as mean±s.e.m. (**d**) Representative images of cells measured in **a** before Tg stimulation. Scale bars, 10 μm.

We used total internal reflection fluorescence (TIRF) microscopy to assess the ability of STIM2.1 to oligomerize, translocate towards the ER–PM junctional regions, to recruit Orai1 to STIM2 punctae and to interact with Orai1. HEK239 cells were co-transfected with Orai1-mEGFP and STIM2.1 or STIM2.2 tagged with mcherry replacing aa 648–711 (see Fig. 3) and treated with 1 μM Tg for ∼15 min to induce store depletion followed by end-point analysis. Figure 5a shows that both STIM2 variants as well as Orai1 formed clusters. Manders coefficient (M1 and M2) analysis was performed on background-subtracted images of cells using the JACoP plugin in Fiji^26^. M1 shows the co-localization of STIM2.1 or STIM2.2 with Orai1, whereas M2 depicts the fraction of Orai1 co-localizing with STIM2.1 or STIM2.2. We observed a small decrease in the fraction of Orai1 co-localizing with STIM2.1 (Fig. 5b, M2 red bar); however, STIM2.1 clearly is still able to co-localize with Orai1 (Fig. 5b, M1 red bar). We also did not observe any differences in mutual co-localization among STIM2 splice variants (Supplementary Fig. 2). In stark contrast, Förster resonance energy transfer (FRET) measurements yielded a very different result: as can be appreciated from the FRET channel images shown in Fig. 5a and quantified in Fig. 5c, STIM2.1 shows a marked defect in the apparent FRET efficiencies when compared with STIM2.2. These results explain the inability of STIM2.1 to activate Orai1, but also indicate that either a second interaction site is unperturbed (leading to co-localization) or that an interaction still exists but that the distance between the fluorescent tags now is too far to measure FRET. Controls and correction factors are described in detail in the Methods section. As shown in Fig. 5d, both STIM2.2 and STIM2.1 formed extensive punctae also without stimulation, as predicted from the lower EF-hand Ca^2+^ affinity of STIM2, pointing towards normal luminal Ca^2+^ sensing and oligomerization mechanisms within STIM2.1. The fact that most of these clusters do not contain Orai1 indicates that either STIM2 is not yet completely unfolded or possibly that additional cytosolic factors prevent extensive Orai1 co-clustering. Nevertheless, the high resting calcium (Fig. 4) with overexpression of STIM2.2 indicates the presence of clusters with functional coupling between Orai1 and STIM2.2.

### STIM2.1 opposes STIM2 and STIM1 function

**Figure 6:**
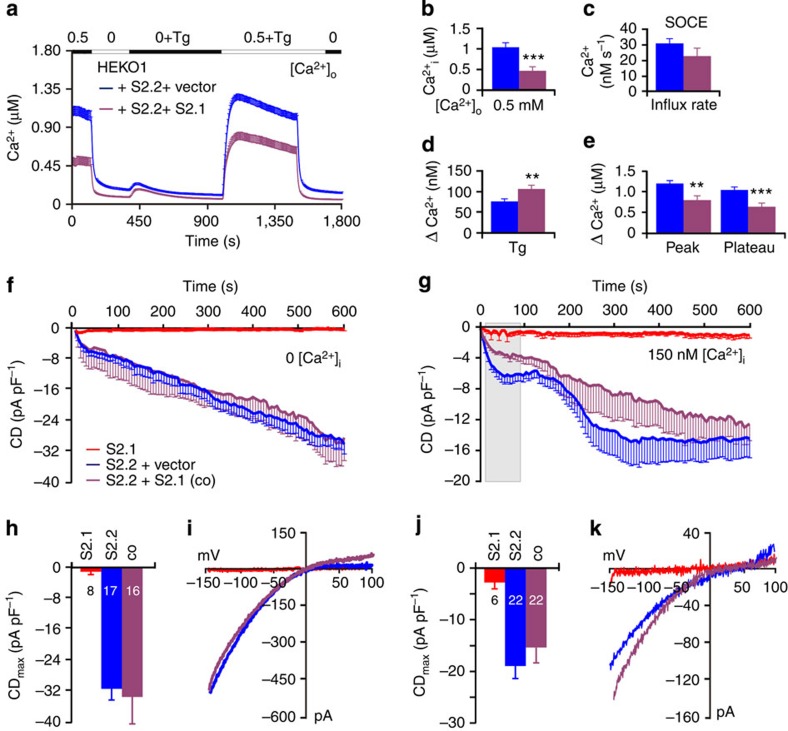
STIM2.1 opposes STIM2.2 function. (**a**) Traces showing average changes in intracellular Ca^2+^ concentration [Ca^2+^]_i_ over time in response to perfusion of different [Ca^2+^]_o_ indicated in the upper bar in HEKO1 cells co-transfected with 0.5 μg CFP-*STIM2.2* together with YFP-control vector (blue) or 1 μg YFP-*STIM2.1* (purple). (**b**–**e**) Quantification of changes in [Ca^2+^]_i_ measured in **a** from 66–69 cells of 6 experiments. (**f**) Average traces showing whole-cell current density (CD) over time extracted at −130 mV in HEKO1 cells co-transfected like in **a** or with 1 μg YFP-*STIM2.1* (red, same trace as Fig. 4f) with internal solution clamping [Ca^2+^] to 0. (**g**) Average traces showing whole-cell CD over time measured from cells transfected like in **f** with an internal solution clamping [Ca^2+^] to 150 nM. Grey box denotes time points between 12 and 90 s after break-in. (**h**,**j**) Average maximum CD recorded from cells measured in **f** and **g**, respectively. (**i**,**k**) Current–voltage (*I*–*V*) relationship of representative cells recorded at maximal current in **f** and **g**, respectively. **P<0.01, ***P<0.001, Student's *T*-test. Data obtained are presented as mean±s.e.m.

Given the results shown in Figs 2 and 3, we also investigated heterologous co-expression and co-transfected HEKO1 cells with *STIM2.1* together with *STIM2.2*, *STIM1* or an equimolar amount of *YFP* containing a control vector. Co-overexpression of STIM2.2 and the control vector in HEKO1 cells results in an extremely high basal Ca^2+^, indicating indeed a high activity of coupled STIM2.2 and Orai1. Nonetheless, STIM2.2 still elicits additional activation of Orai1 after store depletion in 0 mM Ca^2+^ (Fig. 6a–e). Keeping *STIM2.2* constant and co-expressing *STIM2.1* significantly decreased pre-coupled and full-depletion-operated Ca^2+^ (Fig. 6a–e). Analysed cells were not sorted for preactivated cells. We also performed whole-cell patch-clamp recordings of HEKO1 cells co-transfected with either splice variant alone and equimolar vector control or with *STIM2.2* and *STIM2.1*. In contrast to the imaging results, co-expression of STIM2.1 did not dampen STIM2.2-mediated activation of Orai1 in conditions of extreme intracellular Ca^2+^ buffering (20 mM BAPTA; IP_3_) (Fig. 6f,h,i). To more closely mimic the Ca^2+^-imaging condition with only very slightly buffered intracellular Ca^2+^ (Fura2), we clamped the intracellular free Ca^2+^ concentration to 150 nM with addition of IP_3_ and repeated the patch-clamp analysis (Fig. 6g,j,k). Now co-expression showed a significant inhibitory effect in the initial store-operated phase (grey box in Fig. 6g): between 12 and 90 s after break-in, 32 of 41 time points showed significantly (*P*<0.05) reduced current densities (*n*=22 cells for each condition) with co-expression of STIM2.1 and STIM2.2. Expression of STIM2.1 did not elicit currents in either condition. Analyses of maximal current densities and exemplary IV relations are shown in Fig. 6h–j. Primary human CD4^+^ T cells as well as Jurkat T cells used in Figs 2 and 3 express both *STIM1* and *STIM2* (Fig. 1); therefore, we also analysed co-overexpression of STIM2 splice variants with STIM1. Co-expression of STIM2.2 with STIM1 in HEKO1 cells leads to an expected significant increase in basal [Ca^2+^]_i_ (Supplementary Fig. 3a); however, STIM1-mediated store depletion-induced Ca^2+^ entry (green trace) was not much affected by co-expression of STIM2.2 analysed within 24 h after transfection (Supplementary Fig. 3a–e). In contrast, while co-expression of STIM2.1 together with STIM1 did not have a significant effect on basal [Ca^2+^]_i_, it significantly inhibited SOCE (Supplementary Fig. 3a–e) thus confirming a negative regulatory role of STIM2.1 in conditions of full store depletion as also seen for naive CD4^+^ T cells and Jurkat T cells (Figs 2c,g and 3). Whole-cell patch-clamp recordings of HEKS1 cells (stable STIM1 expression), with co-overexpression of Orai1 together with vector only, STIM2.2 or STIM2.1 revealed that in strong buffering conditions (20 mM BAPTA, IP_3_) co-expression of both STIM2.2 and STIM2.1 increased STIM1-mediated Orai1 current densities (Supplementary Fig. 3f–h). Clamping [Ca^2+^]_i_ to 150 nM in HEKO1 cells upon co-expression of STIM1 with STIM2.1 or STIM2.2 showed smaller currents with very variable kinetics, thus potential inhibitory effects may be difficult to detect (Supplementary Fig. 3i–k). However, both sets of patch-clamp results (Fig. 5; Supplementary Fig. 3) confirm a direct or indirect Ca^2+^-dependent effect in STIM2.1's ability to act as an inhibitory subunit of SOCE.

### Homology modelling and protein–protein docking analysis

**Figure 7:**
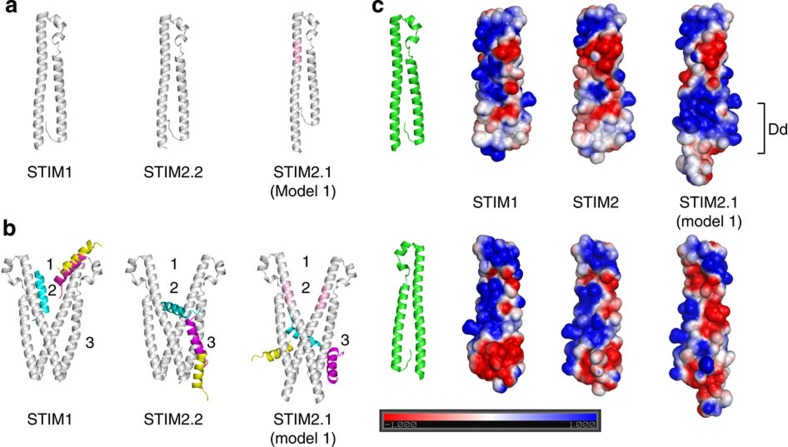
Structural comparison of the STIM CAD domains in the presence or absence of Orai1 C-terminal peptide domain. (**a**) Homology models of the STIM1, STIM2.2 and STIM2.1 CAD domains. The homology models were built according to the template human STIM1 CAD domain (PDB ID: 3TEQ). The inserted segment in the STIM2.1 model is coloured in pink. (**b**) Docking the C-terminal helix of Orai1 on STIM dimers. STIM CAD domains are coloured in grey. The conformations of the C-terminal helix of Orai1 were predicted by the docking packages DOT2 (cyan), FRODOCK (magenta) and ZDOCK (yellow). Motif 1: upper part of CAD domain that is supposed to be close to or in contact with the membrane containing ORAI channel proteins. Motif 2: region near the crossing of STIM CAD dimerization domain. Motif 3: the lateral face of STIM CAD near the dimerization domain. (**c**) Electrostatic potential distribution on the solvent-accessible surfaces of the STIM CAD domains. The unit of electrostatic potential used is kT e^−1^. Dimerization domain (Dd).

To correlate the observed phenotype of STIM2.1 to available structural information on STIM1, we applied homology modelling to predict the structures of STIM2.2 and of STIM2.1 carrying the VAASYLIQ insertion. Homodimers of STIM2.2 and STIM2.1 were modelled according to the structure of the STIM1 dimer (PDB ID: 3TEQ). Details on the applied algorithms and methods as well as some additional validation are given in the Supplementary Material. One certainly needs to acknowledge that, in the case of STIM2.1, the accuracy of modelling such a large insertion by homology modelling is somewhat limited. Thus, we have considered four alternative cases for STIM2.1 (Fig. 7a; Supplementary Fig. a) where the 8 amino-acid insertion leads either to an ideal extension of helix Sα1 (model 1), a flexible loop (model 2) or a partial helix (models 3 and 4). Upon docking these dimer models of the STIM CAD domains to the Orai1 C-terminal helix using the three different docking packages DOT2 (ref. 27), FRODOCK^28^ and ZDOCK^29^, this extension turned out to have a significant, consistent impact on the predicted Orai1-binding sites. For the STIM1 CAD dimer, all best-scoring solutions generated by the packages predicted the Orai1 C-terminal helix to bind to motif 1 of STIM1 (Fig. 7b). In contrast, Orai1 was predicted to favour binding to motifs 2 and 3 of STIM2.2 and of the four STIM2.1 models (Fig. 7b; Supplementary Fig. 4b). Note that due to the symmetric dimer geometry, some configurations docked to the left or right monomer (mostly in motif 3) are in fact (almost) equivalent. On the basis of the proposed model of STIM1–Orai1 coupling reviewed in ref. 13, the C-terminal helix of Orai1 can access the upper part of the CAD domain but hardly is able to approach its lower part, which is close to the dimerization domain (Dd) of CAD. Thus, the postulated docking positions in motifs 2 and 3 are likely not biologically active. To rationalize the docking results, we calculated the electrostatic potential around all CAD domains and the Orai1 C-terminal helix with the software APBS^30^ and plotted the potential on the corresponding solvent-accessible surfaces. As shown in Supplementary Fig. 5, a large fraction of the surface area of Orai1 helix is negatively charged. To make energetically favourable interactions, the Orai1 helix should align its negatively charged surface with the positively charged strip on the STIM1 CAD domain. This is likely the reason why the Orai1 helix was preferably oriented in the upper part of the STIM1 CAD domain termed motif 1 by the three programs (Fig. 7b). Compared with STIM1, the positively charged areas around motif 1 shrink in size in the STIM2.2 and STIM2.1 CAD domains (Fig. 7c; Supplementary Fig. 4c). Besides, in models 2, 3 and 4 of STIM2.1 CAD, the protrusion of the loop (or partial loops) in motif 1 made the interaction surfaces less smooth than the surface of an ideal helix. As a result, alternative positions for the Orai1 helix (near motifs 2 and 3) were predicted to be energetically more favourable for binding to the STIM2.2 and STIM2.1 models (Fig. 7b; Supplementary Fig. 4b). As argued above, these alternative positions are likely biologically not plausible. Figure 7c shows that the electrostatics of the Dd of STIM2.1 model 1 CAD is quite different from that of STIM1 and STIM2.2. Precisely, the Dds of STIM1 and STIM2.2 CAD are relatively neutral, whereas the domains are more positively charged in STIM2.1 model 1. This suggests that STIM2.1 CAD may not function as a stable dimer due to repulsion between its equally charged domains.

### Biochemistry of the STIM2.1 and STIM2.2 CAD domains

**Figure 8:**
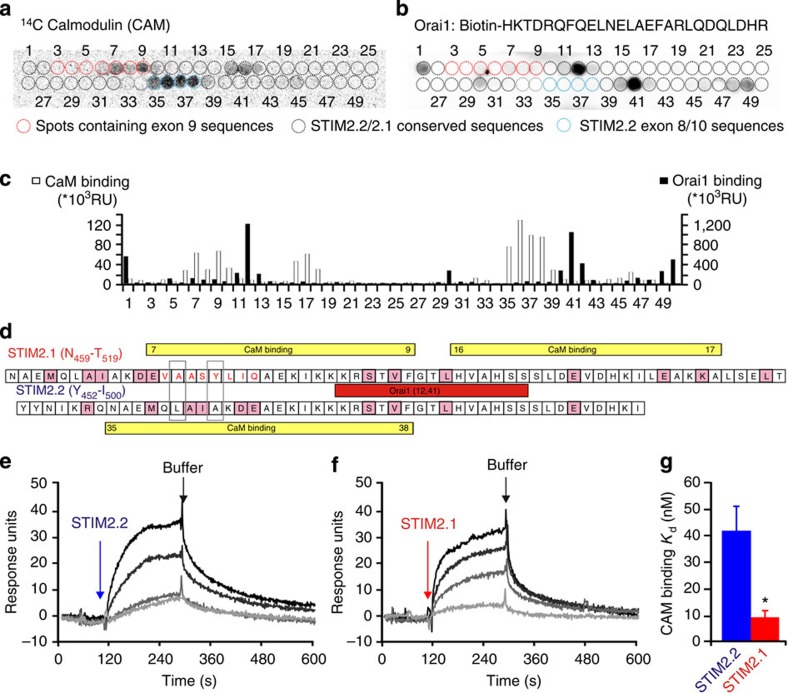
Characterization of Orai1 and Calmodulin-binding properties of STIM2.1 and STIM2.2. (**a**) ^14^C-labelled calmodulin (CaM) binding to STIM2 peptide arrays. (**b**) Biotinylated Orai1 C-term peptide binding to identical STIM2 peptide array as in **a**. Spots encircled with a red dotted line represent peptides encoding exon 9 sequences, blue dotted line represent peptides encoding the sequence flanking exon 9 (exon 8/10 transition unique in STIM2.2) and black circles represent peptides encoding conserved sequence in both splice variants. (**c**) Quantification of bound Orai1 peptide (black bars) or CaM (white bars) to the different peptide spots. (**d**) Schematic representation of partial sequences of STIM2.1 (upper sequence) or STIM2.2 (lower sequence) encoded by the spotted peptides, showing the domains binding to CaM in STIM2.1 (yellow, encoded by peptides 7–9 and 16–17) and in STIM2.2 (yellow, encoded by peptides 35–38) and the identical Orai1-binding domain (red encoded by peptide 12 for STIM2.1 and peptide 41 for STIM2.2). (**e**,**f**) Surface plasmon resonance (SPR) analysis of the binding affinity of calmodulin with representative binding curve sensorgrams showing real association and dissociation of purified CAD domains of STIM2.2 (red-filled boxes denote differences to STIM1). (**e**) or STIM2.1 (**f**) with immobilized CaM. (**g**) Average *K*_d_ values for CaM binding of STIM2.2 (blue) or STIM2.1 (red) obtained from five independent experiments using CAD concentrations ranging from 7.5 to 750 nM, *P<0.05, Student's *T*-test. Data obtained are presented as mean±s.e.m.

To obtain further biochemical evidence of splice variant differences and, as the insertion altered the scores of predicted calmodulin (CaM)-binding sites^31^, we tested the amino acids relevant for binding of the Orai1 C-terminal sequence to both the STIM2.2 and to the STIM2.1 CAD domains by probing immobilized peptide sequences either with ^14^C-labelled glutathione *S*-transferase (GST-CaM) or with a biotinylated Orai1 C-terminal peptide. Each peptide spot contains a 15-amino-acid sequence starting with Y452 (translated from first ATG (NT 268) of NM001169118.1). The subsequent spot started with a three amino-acid shift generating spots with an overlap of 12 identical amino acids (aa) to the next neighbour (Supplementary Fig. 6). Figure 8 shows images of membranes after phosphoimager development (Fig. 8a) or chemiluminescence detection (Fig. 8b). Analysis of resulting signals shows that biotinylated Orai1 binds with high specificity to spot 12 (**KR**STVFGTLHVA**H**SS) present in both splice variants (spot 41 is identical, see Supplementary Methods). Both the preceding spot 11 (IKKKRSTVFGTLHVA) and the following spot 13 (TVFGTLHVAHSSSLD) show nearly no binding, indicating that the presence of K477 and R478 as well as of H489 (highlighted in bold in the above sequence of spot 12) is likely critical for Orai1 binding to STIM2. Binding of CaM shows splice-specific differences: Here inclusion of the 8 aa adds an almost classic CaM-binding motif (**IQ**xxx**KI**xxx**R**xx**V**) present in STIM2.1 spot 9 (L**IQ**AE**KI**KKK**R**ST**V**F). STIM2.2 sequences lack this motif but also bind well to CaM with highest binding to spot 36 (MQLAIAKDEAEKIKK) (Fig. 8c,d). Spots 45–50, derived from a putative *in silico* CaM-binding site located upstream of the transmembrane domain, display only weak CaM binding. Overlay of both Orai1- and CaM-binding motifs on the linear sequence indicates partial overlap of these sites, indicating that binding of either Orai1 or CaM may be mutually exclusive (Fig. 8d). To further analyse CaM binding, we expressed STIM2.1 and STIM2.2 CAD domains in *Escherichia coli*, purified and partially refolded the proteins and binding to CaM with CaM-sepharose pull-down assays in buffers containing Ca^2+^ or EGTA. Supplementary Figure 7 shows that both STIM2 CAD domains bind efficiently to CaM-sepharose in a Ca^2+^-dependent manner (Supplementary Fig. 7a). With subtraction of background (agarose bound) signal, we uncovered a small difference in binding between the variants (Supplementary Fig. 7b). We performed surface plasmon resonance spectroscopy (SPR) to determine relative affinities for binding of CAD domains to immobilized GST-CaM (Fig. 8e–g). No binding was observed in the absence of Ca^2+^, confirming results shown in Supplementary Fig. 7. However, in the presence of Ca^2+^, the STIM2.1 CAD domain shows a significantly higher affinity with a binding constant of 9.1±2.7 nM compared with 40.7±10.5 nM for STIM2.2. These results suggest that STIM2.2 and STIM2.1 are affected by both the local Ca^2+^ and CaM concentration. However, even with a mutated IQ motif, STIM2.1 is unable to activate Orai1 (unpublished data).

## Discussion

In our study, we report the identification and functional analysis of a hitherto unknown STIM2 splice variant, *STIM2.1*, where the sequence VAASYLIQ is spliced into the CAD/SOAR (Sα1) domain. We show that *STIM2.1* is expressed in many cell types, where its relative expression ratio to the known variant STIM2.2 can differ up to eightfold in the cell types we tested. SiRNA-mediated downregulation of *STIM2.1* in primary naive human CD4^+^ cells, which express a high amount of both STIM2 splice variants (Fig. 1), shows that thapsigargin-induced SOCE is increased with no effect on basal Ca^2+^. Given the 44% remaining relative expression level of *STIM2.1* in the siRNA-transfected cells, complete knockout of *STIM2.1* is very likely to display an even stronger amplification of SOCE and may uncover a phenotype on basal [Ca^2+^]_i_. Basal Ca^2+^ levels increase during T-cell activation^32^ and although it is tempting to speculate that this increase in basal [Ca^2+^]_i_ is due to decreased levels of *STIM2.1*, expression of both STIM2.2 and *STIM2.1* decreases after bead contact and remains low in effector cells, whereas STIM1 expression recovers after 48–72 h and now is stronger than the sum of both STIM2 splice variants (Fig. 1c). In naive cells, downregulation of *STIM2.1* is in contrast to the splice-specific downregulation of STIM2.2, which reduced both basal Ca^2+^ as well as SOCE (Fig. 2). Database mining^33^ indicates that CaM is also more highly expressed in naive than in effector CD4^+^ cells. Interestingly, overexpression of both variants in Jurkat T cells (Fig. 3) leads to a slight increase in basal Ca^2+^_i_. The effects upon downregulation (Fig. 2) and overexpression (Figs 3 and 6; Supplementary Fig. 2) suggest that STIM2.1 acts as a negative regulator of both STIM1 and STIM2.2 only in full store depletion-mediated activation of Orai1. The high expression of *STIM2.1* (Fig. 1) in primary CD4^+^ T cells thus may reduce sensitivity towards stimulatory factors and likely aids in maintaining naive cells in an inert state. The inhibitory effect of co-expression in Ca^2+^-imaging experiments is not apparent in whole-cell patch-clamp recordings when cells are dialysed with high amounts of BAPTA, although single STIM2.1 expression is still unable to activate Orai1 in such conditions (Fig. 4), consistent with its lack of interaction seen in FRET experiments (Fig. 5). Homology modelling suggests that STIM2.1 shows altered binding configurations and may not form stable CAD dimers due to electrostatic repulsion of its Dds (Fig. 7). Clamping cytosolic Ca^2+^ to 150 nM in presence of IP_3_ unmasked a dominant-negative effect of STIM2.1 on the store-operated phase of STIM2.2-mediated currents and abolished amplification of STIM1 currents (Supplementary Fig. 2i–k). Together, these results suggest that either heterodimers of STIM2.1 with STIM2.2 and possibly with STIM1 can become functional in conditions of extreme buffering (high BAPTA, 0 [Ca^2+^]) or that STIM2.1 precludes binding of STIM2.2 or STIM1 to Orai1 subunits by steric hindrance only if cytosolic Ca^2+^ ions are present, the latter we believe to be more likely. A differential Ca^2+^ requirement for steric hindrance may explain why STIM2.1's inhibitory effect on basal Ca^2+^ is not always seen upon overexpression (compare basal Ca^2+^ of Figs 3 and 4 with Fig. 6) and would also indicate a clear dependency on its expression level. Direct binding of Ca^2+^ to the inhibitory domain of STIM1 has been reported^34^, and local cytosolic Ca^2+^ elevations are required for STIM1 deoligomerization and termination of store-operated Ca^2+^ entry^35^, but it is unclear how cytosolic Ca^2+^ affects STIM2 oligo- or deoligomerization. Parvez *et al*.^10^ showed that STIM2 shows a biphasic activation of Orai1 with an early store-dependent phase and a slower and store-independent activation due to loss of CaM through intracellular dialysis (see also Fig. 4). Our comparison of STIM2.1 and STIM2.2 CAD domain binding to CaM using SPR shows that both domains bind to Ca^2+^-CaM with a much higher affinity than the C-terminal site (10 and 40 nM versus ∼1 μM^36^,^37^). STIM2.1 has an increased affinity and an altered CaM-binding motif likely generated in part by the specific aa sequence (IQ). We also show that Orai1- and CaM-binding sites overlap on a linear peptide array, giving biochemical evidence to the hypotheses of Parvez *et al*.^10^ that CaM binding to STIM2 can prevent its activation of Orai1. However, the fact that STIM2.1 by itself is unable to activate Orai1 even after long dilution of cytosolic factors in the presence of BAPTA, or upon mutation of its IQ motif (unpublished data) argues against CAM binding and permanently occluding the Orai1-binding site. STIM2.1 can still clearly co-localize with Orai1 upon full store depletion (Fig. 5), but the tight interaction with Orai1 required for gating is impaired (FRET). The reduced interaction may be caused by the electrostatic destabilization of STIM2.1 dimer formation and of the Orai1-binding site predicted by molecular modelling analyses (Fig. 7).

Because basal [Ca^2+^]_i_ is significantly increased with expression of STIM2.2 in HEKO1 cells independent of the nature of its signal peptide (short STIM1 or long STIM2), it is highly unlikely that this constitutive entry is mediated by non-ER-localized STIM2 as postulated by Graham *et al*.^9^. Besides the steric and electrostatic implications derived from our molecular modelling approach (Fig. 7), the insertion of VAASYLIQ can also be compared with the effects of mutating amino acids in a similar upstream position relative to the Orai1-binding site in the STIM1 CAD domain: a STIM1 A376 (corresponding to STIM2.2 A467=Y in STIM2.1, see box 1 in Fig. 8d) to K mutant constitutively forms punctae at resting ER Ca^2+^ with no ability to recruit Orai1 to these ER–PM sites^38^. Moreover, a STIM1 L373 (=L464 in STIM2.2, =A in STIM2.1 see box 2 in Fig. 8d) to S mutant fails to couple and activate Orai1 currents^39^. Insertion of VAASYLIQ thus displays a phenotype that is comparable to certain STIM1 mutants within the Sα1 helix of SOAR/CAD. In addition, a very recent report^40^ describes the interaction between CC1 domain preceding the CAD domain and CAD CC3 domain of STIM1 as a critical regulatory mechanism affecting exposure of the CAD CC2 domain, which may also be affected in the case of STIM2.1.

The identification of a STIM2 splice variant antagonizing STIM function resets the stage for analysis of contradictory results regarding the physiological role of STIM2 (that is, in cancer and autoimmunity^14^) as all studies involving downregulation or knockout would affect both variants, thus dampening a ‘true' STIM2.2-mediated effect.

## Methods

### Cell culture and antibodies

All cells were maintained in a 37 °C, 5% CO_2_ humidified incubator in corresponding medium, namely, minimum essential medium for HEK293 WT (HEK) and stably expressing Orai1 (HEKO1), AMIV for human CD4+ cells and RPMI 1640 for E6.1 Jurkat T (Jurkat) cells. All media were supplemented with 10% fetal calf serum and 1% penicillin–streptomycin and HEKO1 were maintained in 1 μg ml^−1^ puromycin. HEK cells were passaged by treatment with trypsin/EDTA. For transfection, the indicated amount of DNA was electroporated into HEK cells and Jurkat cells with Nucleofector II or into CD4+ cells with 4D Nucleofector core unit according to the manufacturer's instructions 16–20 h before measurements. Human *Orai1*, *Orai2* and *STIM1* were subcloned into the EcoRV site of either RFP pMAX, or IRES-RFP-pCAGGS. *STIM2.2* was purchased with an N-terminal CFP or YFP tag (Addgene). We used this cDNA (*STIM2.2*: pEX-CMV-SP-YFP-*STIM2*) and two complementary primers (see Supplementary Table 1) each encoding part of the exon 9 sequence to insert the desired nucleotides into the exon 8/10 boundary. All constructs were confirmed by sequencing. Antibodies used in this study were anti-His Tag (Cell Signaling, 2366, 1:1,000); anti-STIM2 (C-term, Sigma, #S8572, 1:2,000); anti-Calnexin (Stressgen, #SPA-865, 1:1,000) and anti-γ-tubulin (Cell Signaling, #5886, 1:1,000).

### Quantitative real-time PCR

For qRT–PCR, the indicated cell types were harvested in TRIzol (Life Technologies) and stored at −80 °C until RNA was isolated following the manufacturer's instructions. SuperScriptTMII Reverse Transcriptase (Life technologies) was used to generate cDNA and subsequent PCR or qRT–PCR was conducted using the QuantiTect SYBR Green Kit (Qiagen) and a CFX96 Real-Time System (Bio-Rad) with the primers listed in Supplementary Table 1. For quantification, expression levels are presented as the normalized quantification cycle (Cq) values of the gene of interest to that of TBP (TATA box-binding protein) using the ΔCT method (results were comparable when normalized to RNA polymerase).

### Small interfering RNA knockdown

Splice-specific siRNA targeting exon 9 or exon 8/10 boundary were used to specifically knock down endogenous *STIM2.1* or *STIM2.2*, respectively. Using electroporation as mentioned above, 8 μl of 40 μM stock solution of the indicated siRNA or an equivalent concentration of non-targeting RNA was transfected in parallel for the non-silencing control were transfected into naive CD4+ cells. Transfection was repeated on the next day and measurements were conducted 14–18 h later. The sequences of the siRNA are listed below. Knockdown efficiency and off-target effects were tested by qRT–PCR, where the mRNA level was normalized to TBP. Sequences of the used siRNA and qRT–PCR primers are listed in Supplementary Table 1.

### Electrophysiology

Recordings were performed at room temperature in the tight-seal whole-cell configuration, and linear voltage ramps from −150 to +150 mV were applied as in ref. 6. The pipette solution contained the following (in mM): 120 Cesium-glutamate, 3 MgCl_2_, 20 Cesium-BAPTA, 10 Hepes and 0.05 IP3 (pH 7.2 with CsOH). Where indicated, internal solution contained 150 nM free Ca^2+^. The external solution contained (in mM): 120 NaCl, 10 TEA-Cl, 10 CaCl_2_, 2 MgCl_2_, 10 Hepes and glucose (pH 7.2 with NaOH).

### Fluorescence-based Ca^2+^ imaging

Human CD4^+^ or Jurkat T cells were loaded in suspension with 1 μM Fura 2-AM at room temperature for 25 min and seeded on poly-ornithine-coated glass coverslips. HEKO1 cells were loaded at 37 °C for 25 min with slight rotation on an orbital shaker. All experiments were performed using a self-built perfusion chamber with low volume and high solution exchange rate at room temperature. The external Ca^2+^ Ringer solution contained (in mM): 155 NaCl, 2 MgCl_2_, 10 glucose, 5 Hepes and 0.5 CaCl_2_ (0.5 Ca^2+^ Ringer) or no CaCl_2_, but 1 EGTA and 3 MgCl_2_ instead (0 Ca^2+^ Ringer) (pH 7.4 with NaOH). Images were analysed with TILLVision software. The absolute intracellular Ca^2+^ concentration was estimated from the relation [Ca^2+^]_i_=K*(*R*−*R*_min_)/(*R*_max_−*R*) where the values of *K*, *R*_min_ and *R*_max_ were determined from an *in situ* calibration of Fura 2-AM in Jurkat T cells as described in ref. 41. Quantification of the trace shows the average basal [Ca^2+^], the maximal Tg-induced peak in [Ca^2+^]_i_, maximal and plateau [Ca^2+^]_i_ upon readdition of 0.5 mM [Ca^2+^]_o_ and influx rate of cells. The minimal [Ca^2+^]_i_ before addition of Tg or 0.5 mM [Ca^2+^]_o_ was subtracted from Tg-peak or maximal [Ca^2+^]_i_, respectively.

### TIRF microscopy

HEK293 were co-transfected with 6 μg *STIM2.1*-mcherry or *STIM2.2*-mcherry-pIRES and 2 μg Orai1-GFP-pMAX and seeded on 25-mm glass coverslips 24 h before measurement. Stores were depleted by incubation with 1 μg Tg in 0 Ca^2+^ Ringer solution for 15 min. A Leica AM TIRF MC system was used as in ref. 6. The TIRF focal plane was set to acceptor fluorescence and three sets of images (green fluorescent protein (GFP), FRET and mcherry) were captured: GFP was excited using a 488-nm laser (suppression filter BP 525/50); for mcherry, the laser excitation wavelength was 561 nm (suppression filter BP 600/40) and for FRET image, a 488-nm laser was used (suppression filter BP 600/40). Image acquisition and analysis were performed with LAS (Leica Application suite) FRET module. Acquisition parameters (laser intensity 40%, exposure time 100 ms and penetration depth 200 nm) were held constant for all three channels. The apparent FRET efficiency (*E*_A_) was calculated from background-subtracted images using 
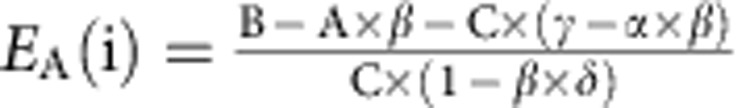
 described by Van Rheenen^42^, where A, B and C stand for donor-, FRET- and acceptor channel, respectively. Bleed through and crosstalk factors (*α*, *β*, *γ* and *δ*) were determined individually for every experimental day using single transfected cells. To ensure that FRET was compared for identical acceptor to donor ratios, only FRET values of regions of interest enclosing STIM2 clusters, which were within average acceptor to donor ratios±one s.d. of the respective experimental day, were used for final analysis.

### Expression and purification of the CAD domains

The cDNA coding for CAD domain of human *STIM2.2* or *STIM2.1* were amplified using the above mentioned pEX plasmids with primers including *Bam*HI and *Xma*I recognition sites and subcloned into a self-modified pET19b vector creating an N-terminal 6 His tag. Plasmids were expressed in *E. coli* BL21 Rosetta strain. Overnight cultures were inoculated in 2 × YT medium containing 0.4% glucose and on the next day diluted 1:20, grown to an OD_600_ of 0.5 before protein production was induced with 1 mM isopropyl-b-D-thiogalactoside for 6 h at 30 °C. Lysis and protein purification were done as in ref. 43. After purification of the His-tagged domains, excess urea and imidazole were removed using illustra NAP-5 Columns (GE Healthcare), and protein was eluted in buffer containing 500 mM NaCl, 50 mM Tris and 0.1% Triton X-100, pH 7. Fractions of the protein were collected and protein concentration was determined using the BCA protein assay kit (Pierce).

### Homology modelling and protein–protein docking analyses

Full details of STIM2 homology modelling and protein–protien docking analyses is given in Supplementary Tables 2–4, Supplementary Figs 8 and 9 and Supplementary Methods.

### Surface plasmon resonance analysis

SPR spectroscopy was carried out in a BIAlite X system. Briefly, monoclonal goat anti-GST serum (BIACORE, Freiburg, Germany) was immobilized on a CM5 research grade sensor chip (BIACORE) by amine coupling according to the manufacturer's instructions. The chip was equilibrated with application buffer (10 mM HEPES pH 7.4, 150 mM NaCl, 6.4 KCl, 2 mM MgCl_2_ and 0.005% P20) at a flow rate of 20 μl min^−1^. GST-CaM was bound to the immobilized antibodies in the measuring cell. Similarly, immobilized GST in the reference cell served as a negative control. Subsequently, solutions containing increasing concentrations of purified STIM2.1 and STIM2.2 CAD domains (7.5–750 nM) were passed over the chip in the presence of 2 mM Ca^2+^. Each STIM application was followed by application of running buffer. The analysis was carried out employing the BIA evaluation software version 3.1 (BIACORE).

### Peptide-spot-binding assay

Peptides encoding for STIM2 CAD domains were synthesized on acid-hardened cellulose membranes, derivatized with a polyethylene glycol spacer as described^44^. Membranes were equilibrated in binding buffer (150 mM NaCl, 50 mM Tris/HCl (pH 7.5), 0.1% Triton X-100 and 1 mM CaCl_2_ or 4 mM EGTA) for 2 h at 4 °C. ^14^C-labelled GST-CaM was added and incubated at 4 °C overnight. The membrane was washed with binding buffer three times for 10 min each, dried at room temperature and subjected to phosphorimaging using a Typhoon-Trio imaging device (GE Healthcare). An equivalent peptide filter was incubated overnight with 10 μM biotinylated peptide in TBS with 5% biotin-free Albumin fraction V and 2.5% saccarose after blocking in buffer only for 3 h at room temperature. Blots were washed and developed after incubation with Avidin-HRP and subsequent chemiluminescence detection.

### Calmodulin pull-down assay

The purified STIM2.1 and STIM2.2 CAD domains were incubated at a final concentration of 2.5 μM in total volume of 250 μl with 25 μl prewashed CaM-sepharose beads (GE Healthcare) for 1 h at 4 °C with overhead rotation. The binding buffer contained 25 mM Tris, pH 7.5, 150 mM NaCl and 1 mM CaCl_2_ or 1 mM EGTA. Unbound fraction was removed by washing three times with the corresponding buffer and bound fraction was eluted by boiling at 65 °C for 15 min with 2 × Laemmli buffer and finally analysed by electrophoresis. The western blot was probed with anti-His antibody (Cell Signaling) at 1:1,000 dilution. Uncropped versions of al blot and gel images are shown in Supplementary Fig. 10.

## Author contributions

A.-M.M., D.A. and B.A.N. conceived the project, designed the experiments and wrote the manuscript. A.-M.M. and D.A. performed the experiments and analysed the data. G.S. contributed the qRT–PCR analysis and gave technical support. P.-H.L. and V.H. undertook homology modelling and protein–protein docking analyses. M.J. designed peptide blots and performed radioactive experiments.

## Additional information

**How to cite this article:** Miederer, A.-M. *et al*. A STIM2 splice variant negatively regulates store-operated calcium entry. *Nat. Commun.* 6:6899 doi: 10.1038/ncomms7899 (2015).

## Supplementary Material

Supplementary Information Supplementary Figures 1-10, Supplementary Tables 1-4 and Supplementary Methods and Supplementary References
