# Supplementary material for: A STIM2 splice variant negatively regulates store-operated calcium entry
Source: Nat Commun. 2015 Apr 21;6:6899. doi: 10.1038/ncomms7899 (PMC4411291; doi:10.1038/ncomms7899)
Supplement: Supplementary Information — Supplementary Figures 1-10, Supplementary Tables 1-4 and Supplementary Methods and Supplementary References [file ncomms7899-s1.pdf]

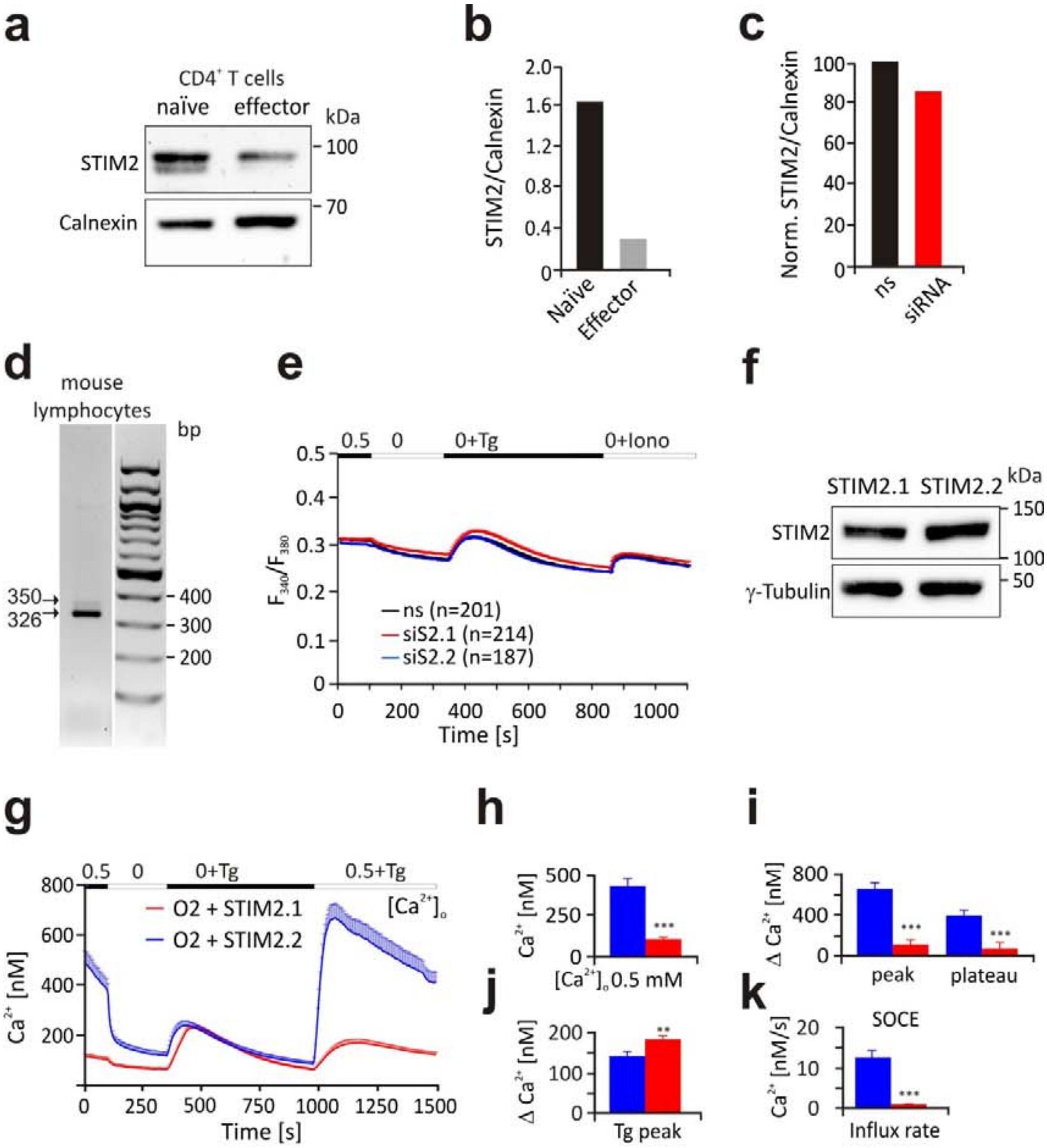

Supplementary Figure 1 | (**a**) Immune blot of lysates of naïve (left lane) and 72h anti- CD3/anti-CD28 bead stimulated CD4+ cells (right lane). Blot was probed with anti STIM2 and with anti Calnexin as input control. (**b**) Bar graphs showing STIM2 normalized to calnexin. (**c**) Quantification STIM2 of CD4+ naïve T cells transfected with control (black, ns) or siRNA against STIM2.1(red, siRNA) where the ratio of STIM2 to calnexin of control treated cells was set to 100 %. (**d**) Image showing PCR amplification products obtained with flanking primer pair (Supplementary Table 1) using cDNA from mouse lymphocytes (**e**)Traces showing average changes in intracellular Ca^{2+} concentration $[Ca^{2+}]_i$ over time in response to perfusion of different $[Ca^{2+}]_o$ indicated in the upper bar or in response to application of 5μM ionomycin in CD4+ T cells transfected with nonsilencing RNA (black), or splice specific siRNA silencing STIM2.1 (red) or STIM2.2 (blue) (**f**) Western blot showing heterologous expression of STIM2 (upper panel) relative to γ-tubulin(lower panel) in HEKO1 cells transfected with YFP-STIM2.1 (left) or YFP-STIM2.2 (right) (**g**) Traces showing average changes in intracellular Ca^{2+} concentration $[Ca^{2+}]_i$ over time in response to perfusion of different $[Ca^{2+}]_o$ indicated in the upper bar in HEKWT cells co-transfected with Orai2 IRES RFP and YFP-STIM2.1 (red) or STIM2.2 (blue). (**h-k**) Quantification of changes in $[Ca^{2+}]_i$ measured in **f**. Asterisks indicate *p<0.05, **p<0.01, ***p<0.001.Student T-test. 5-8 individual experiments.

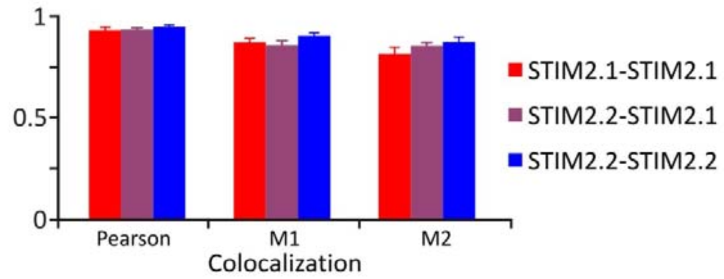

Supplementary Figure 2 | Colocalization analyses of STIM2 splice variants using Pearson and Manders coefficients (M1) or (M2) for image analysis. Constructs containing mcherry or EGFP replacing I648-K711 were used.

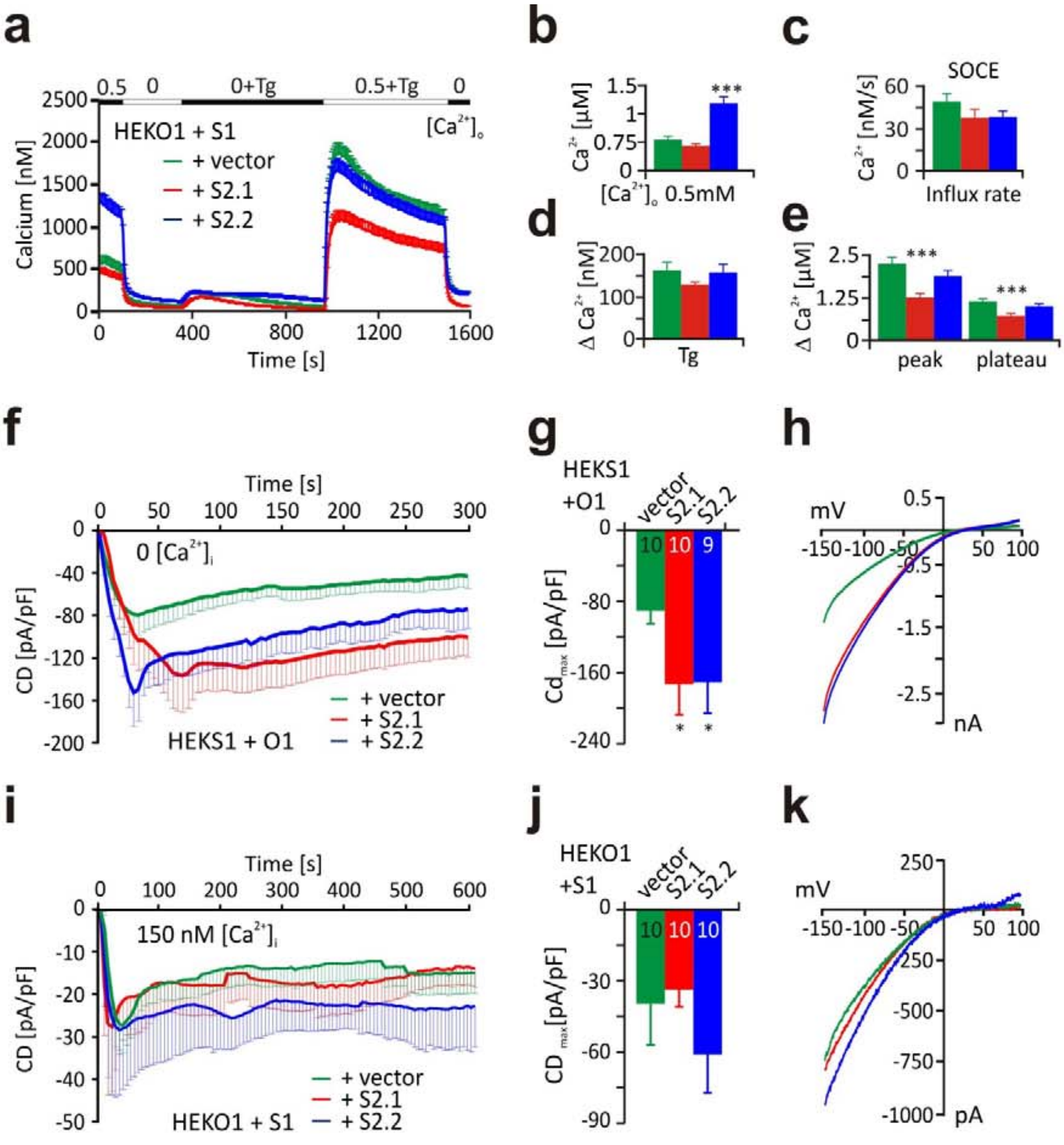

Supplementary Figure 3 | STIM2.1 affects STIM1 function (**a**) Traces showing average changes in intracellular Ca^{2+} concentration $[Ca^{2+}]_i$ over time in response to perfusion of different $[Ca^{2+}]_o$ indicated in the upper bar in HEKO1 cells co-transfected with 1µg STIM1-IRES-RFP together with 1 µg YFP-STIM2.1 (red) or 1µg STIM2.2 (blue) or a control vector (green). (**b-e**) Quantification of changes in $[Ca^{2+}]_i$ measured in **a** from 95-125 cells from 8-9 individual experiments. (**f**) Average traces showing whole cell current density (CD) over time extracted at -130mV with the internal solution containing 20 mM BAPTA (0 $[Ca^{2+}]_i$) in HEKS1 cells co-transfected with 1µg Orai1-IRES-RFP together with 1µg YFP-STIM2.1 (red) or 1µg YFP-STIM2.2 (blue) or control vector (green). (**g**) Average maximum CD recorded from cells measured in **f**. (**e**) Current-voltage (I-V) relationship of representative cells recorded in **f**. (**i**) Average traces showing whole cell current density (CD) over time extracted at -130mV with the internal solution containing 150 nM $[Ca^{2+}]_i$ in HEKO1 cells transfected as in **a**. (**j**) Average maximum CD recorded from cells measured in **i**. (**k**) Current-voltage (I-V) relationship of representative cells recorded in **f**. Only significantly different values, tested against HEKO1 + S1 + vector, are indicated. Asterisks indicate significant differences using unpaired, two-tailed Student T-test with *$p<0.05$, **$p<0.01$, ***$p<0.001$.

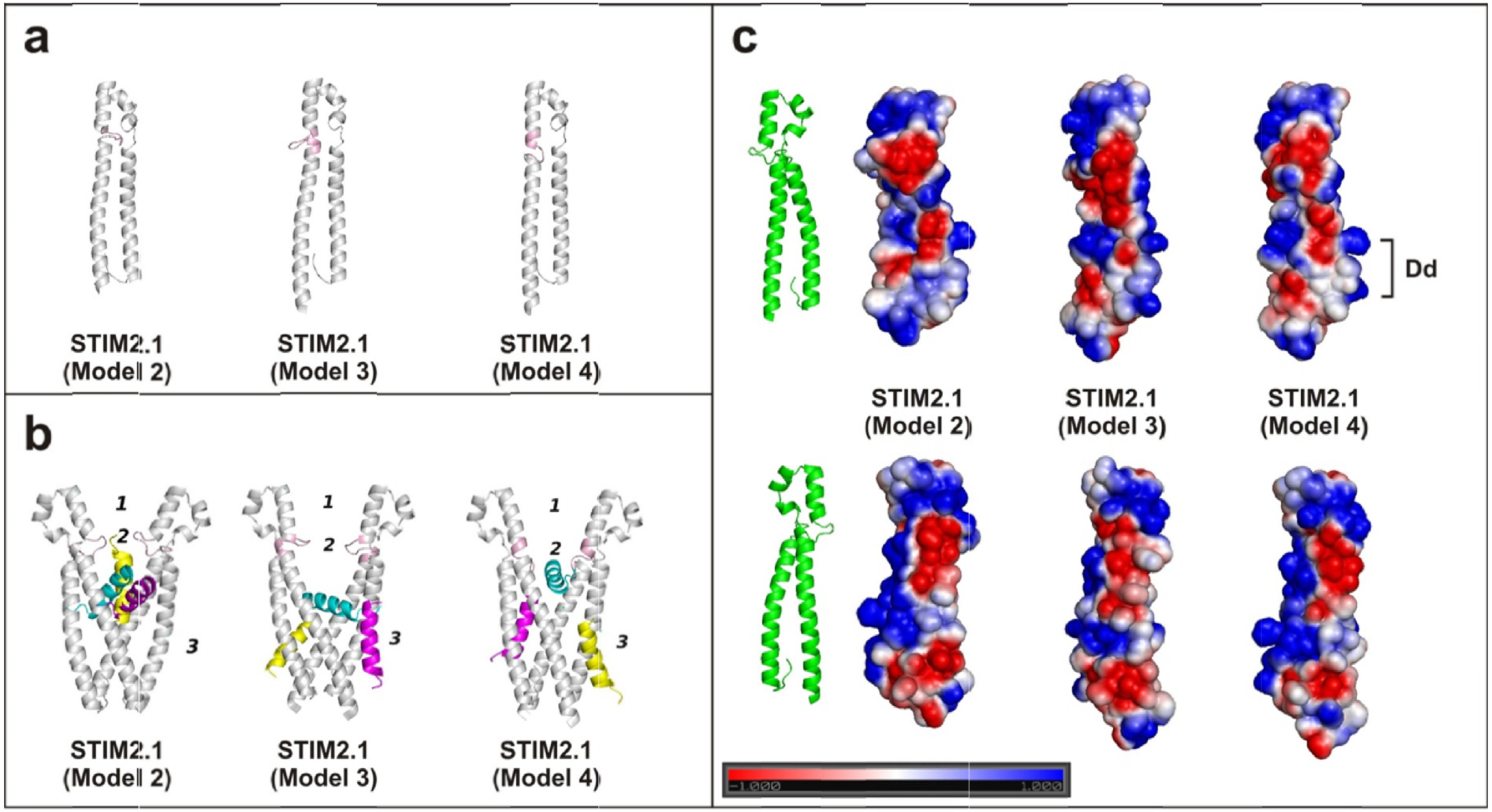

Supplementary Figure 4. Structural comparison of the STIM2.1 CAD domains in the presence or absence of the Orai1 C-terminal peptide domain (a) Homology models of the STIM2.1 CAD domains. The homology models were built according to the template human STIM1 CAD domain (PDB ID: 3TEQ). The inserted segments in the STIM2.1 models are colored in pink. (Details of this are given in the Supplementary methods). (**b**) Best-scoring docking conformations for the C-terminal helix of Orai1 on STIM2.1 dimers. STIM2.1 CAD domains are colored in grey. The conformations of the C-terminal helix of Orai1 were predicted by the docking packages DOT2 (cyan), FRODOCK (magenta) and ZDOCK (yellow). We classified the conformations as Motif 1: upper part of CAD domain that is supposed to be close to or contact to the membrane containing ORAI channel proteins; Motif 2: region near the crossing of STIM CAD dimerization domain; and Motif 3: The lateral face of STIM CAD near the dimerization domain. (**c**) Electrostatic potential distribution on the solvent accessible surfaces of the STIM2.1 CAD domains. The unit of electrostatic potential used is kT/e. Dimerization domain (Dd).

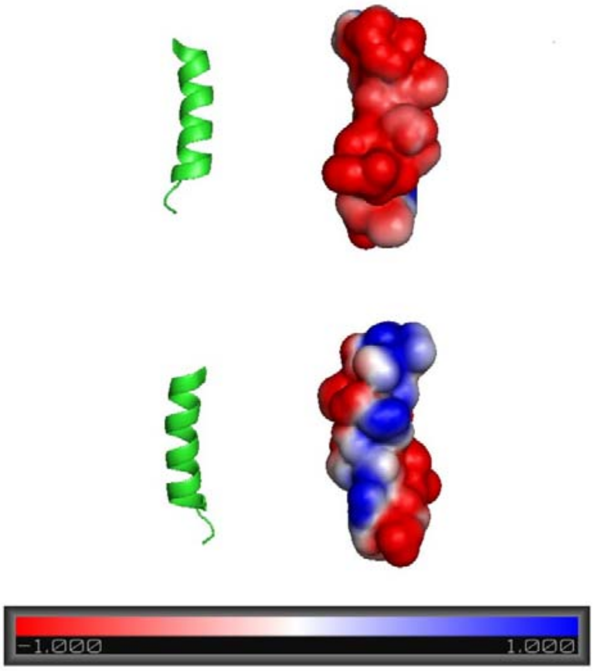

Supplementary Figure 5 | Electrostatic potential distribution on the solvent accessible surface of the C-terminal domain of Orai1. The unit of electrostatic potential is kT/e.

STIM2.1 Y452 - W559

1. YYNIKRQNAEMQLAI
2. IKRQNAEMQLAIAKD
3. QNAEMQLAIAKDEVA
4. EMQLAIAKDEVAASY
5. LAIAKDEVAASYLIQ
6. AKDEVAASYLIQAEK
7. EVAASYLIQAEKIKK
8. ASYLIQAEKIKKKRS
9. LIQAEKIKKKRSTVF
10. AEKIKKKRSTVFGTL
11. IKKKRSTVFGTLHVA
12. KRSTVFGTLHVAHSS
13. TVFGTLHVAHSSSLD
14. GTLHVAHSSSLDEVD
15. HVAHSSSLDEVDHKI
16. HSSSLDEVDHKILEA
17. SLDEVDHKILEAKKA
18. EVDHKILEAKKALSE
19. HKILEAKKALSELTT
20. LEAKKALSELTTCLR
21. KKALSELTTCLRERL
22. LSELTTCLRERLFRW
23. LTTCLRERLFRWQQI
24. CLRERLFRWQQIEKI
25. ERLFRWQQIEKICGF
26. FRWQQIEKICGFQIA
27. QQIEKICGFQIAHNS
28. EKICGFQIAHNSGLP
29. CGFQIAHNSGLPSLT
30. QIAHNSGLPSLTSSL
31. HNSGLPSLTSSLYSD
32. GLPSLTSSLYSDHSW

STIM2.2 N454-I500

33. NIKRQNAEMQLAIAK
34. KRQNAEMQLAIAKDE
35. NAEMQLAIAKDEAEK
36. MQLAIAKDEAEKIKK
37. AIAKDEAEKIKKKRS
38. KDEAEKIKKKRSTVF
39. AEKIKKKRSTVFGTL
40. IKKKRSTVFGTLHVA
41. KRSTVFGTLHVAHSS
42. TVFGTLHVAHSSSLD
43. GTLHVAHSSSLDEVD
44. HVAHSSSLDEVDHKI

STIM2 E262 - W301

45. EPSFMISQLKISDRS
46. ISQLKISDRSHRQKL
47. ISDRSHRQKLQLKAL
48. HRQKLQLKALDVVLF
49. QLKALDVVLFGPLTR
50. DVVLFGPLTRPPHNW

Supplementary Figure 6 | Sequence of peptide spot array. Peptides were designed to cover sequences encoding CAD domains of STIM2.1 and STIM2.2 with each peptide encoding 15 amino acids with a 3 amino acid frame shift in the next spot. The sequence encoding exon 9 is highlighted in red in peptides 3-9. STIM2.1 CAD domain is covered by peptides 1-32 while the conserved sequence in STIM2.2 is covered by peptides 33-44. The identical peptides are enclosed with a dotted box. Other peptides representing exon 8-10 transition are STIM2.2 unique. Peptides 45-50 encode a predicted CaM binding site located upstream of the transmembrane domain.

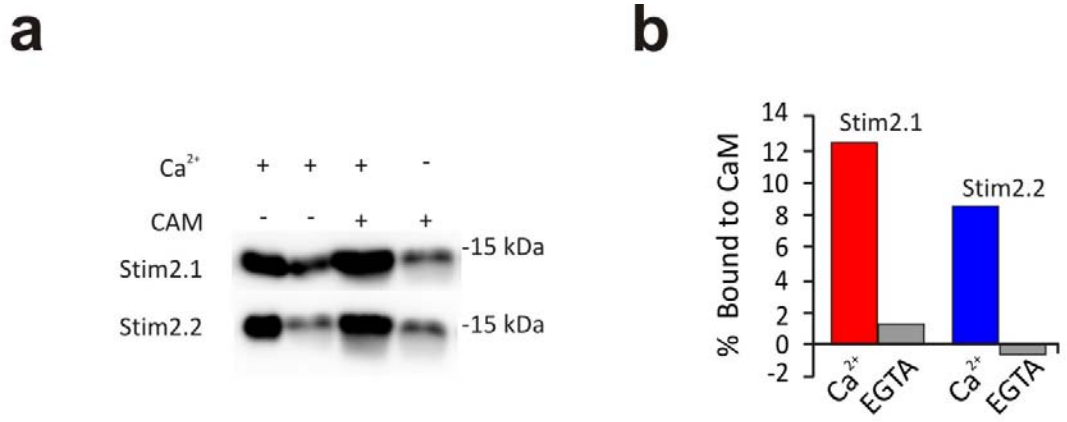

Supplementary Figure 7 | CaM pull down assay (**a**) Immune blot showing (from left to right) input control, fraction of bound CAD domain to agarose beads in absence of CaM, CaM agarose-bound fraction in presence of 1 mM Ca^{2+} or in absence of Ca^{2+} for STIM2.1 (upper panel) or STIM2.2 (lower panel). Blot was probed with anti His antibody. (**b**) Quantification of the bound fractions in presence of Ca^{2+} of CAD domains of STIM2.1 (red) and STIM2.2 (blue) or the corresponding bound fractions in absence of Ca^{2+}. The shown bound fractions are corrected for unspecific background binding by subtracting the fractions bound to agarose beads in absence of CaM

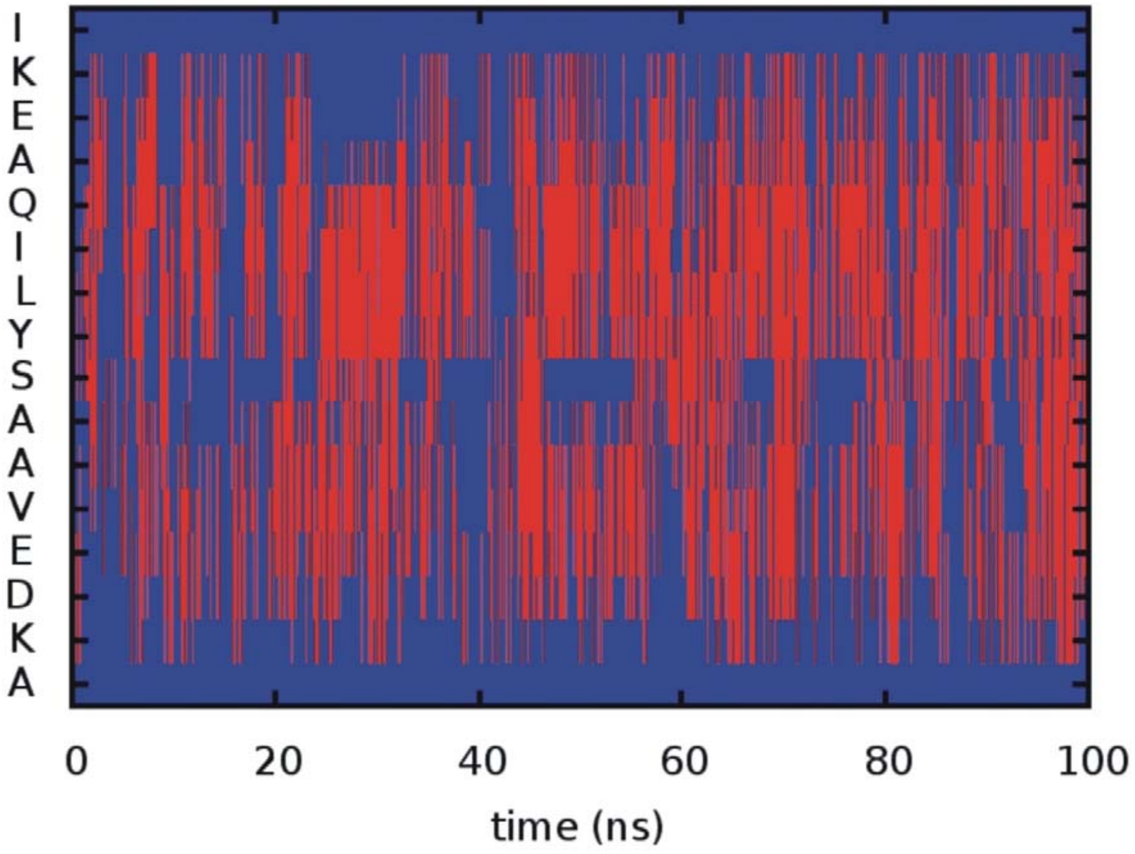

Supplementary Figure 8 | Secondary structure for the 16-residue peptide AKDEVAASYLIQAEKI during a 100 ms long self-guided Langevin dynamics stimulation. Red color marks the time points when the backbone of a particular residue adopted an alpha-helical conformation (according to the DSSP program) whereas blue markus non-helical regions.

```
>P1;STIM2_1
sequence:STIM2_1:435 : 542: STIM2_1:human::

PDALQKWLQLTHEVEVQYYNIKRQNAEMQLAIAKDEVAASYLIQAEKIKKKRSTVFGTLHVAHSSSL
DEVDHKILEAKKALSELTTCLRERLFRWQQIEKICGFQIAH*

>P1;STIM1_SOAR
structureX:3TEQ_A.pdb:344A:443:A:STIM1 SOAR:human::
--------PEALQKWLQLTHEVEVQYYNIKKQNAEKQLLVAKEGAEKIKKKRNTLFGTFHVAHSSSL
DDVDHKILTAKQALSEVTAALRERLHRWQQIEILCGFQIVN*

>P1;STIM1_extend
structureX:3TEQ_A_ext.pdb:344A:443:A:STIM1 SOAR:human::
PEALQKWLQLTHEVEV---------------------------------------------------
-----------------------------------------*
```

Supplementary Figure 9 | Multiple template scheme to build STIM2.1 model 1.

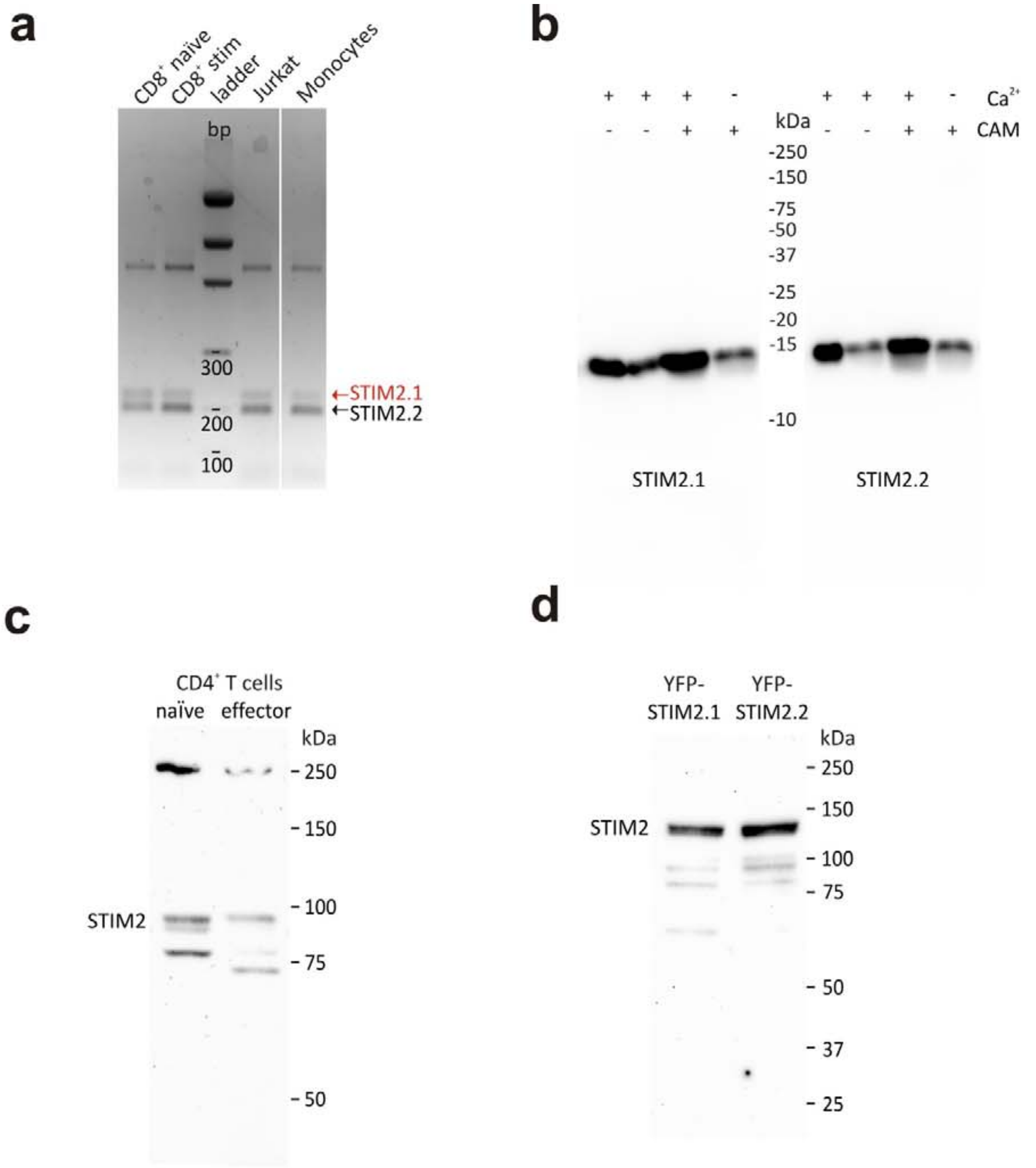

Supplementary Figure 10 | (**a**) uncropped agarose gel of Fig. 1b, (**b**) uncropped immune blot of Fig. S4a (**c**) uncropped immune blot of Fig. S1a. (**d**) uncropped immune blot of Fig. S1e.

Flanking *h.s.* STIM2 exon 9 (for)	GGCTCTGAAAAAGGCCGAA
Flanking *h.s.* STIM2 exon 9 (rev)	GTCCCAAAGACTGTGCTTCTC
qRTPCR primer for	CTCTGAAAAAGGCCGAAAAA
qRTPCR primer rev +exon9	TGAAGCAGCAACCTCATCTTT
qRTPCR primer rev -exon9	TTTTCTGCCTCATCTTTAGCAA
core siRNA sequence +exon9	GAUGAGGUUGCUGCUUCAU
core siRNA sequence -exon9	AGAUGAGGCAGAAAAAAUU
Exon 9 cloning primer for	ATCTGATTCAGGCAGAAAAAATTAAAAAGAAGAGAAGC
Exon 9 cloning primer rev	ATGAAGCAGCAACCTCATCTTTAGCAATAGCTAGCT
Flanking *m.m.*. STIM2 exon 9 (for)	CCAAGGAGGAGGCCTGTC
Flanking *m.m.* STIM2 exon 9 (rev)	GGTCTACTTCGTCCAGGG
EGFP/mcherry/YFP for Pho	ATGGTGAGCAAGGGCGAG
EGFP rev with two extra NT, Pho	ATCTTGTACAGCTCGTCCATG

Supplementary Table 1. Primer and siRNA sequences

		STIM1	STIM2.2	STIM2.1			
				Model 1	Model 2	Model 3	Model 4
QMean6		0.676	0.690	0.564	0.557	0.547	0.522
Procheck	Core	0.951	0.948	0.981	0.942	0.923	0.942
	Allow	0.049	0.052	0.019	0.048	0.077	0.048
	Generous	0	0	0	0	0	0.010
	Disallow	0	0	0	0.010	0	0

Supplementary Table 2. Qmean6 scores and stereochemistry evaluations for the X-ray structure of STIM1 and for all STIM2 models.

Case study				Docking methods RMSD$_{CA}$ (Å)		
Protein complex	PDB ID	Receptor	Ligand	DOT2	FroDOCK	ZDOCK
SMRT/GPS2	2L5G	SMRT	GPS2	0.29	0.24	0.77
MST1/RASSF5 SARAH domain	4OH8	MST1	RASSF	0.50	0.31	0.66
BRCA1/BARD1	1JM7	BARD1	BRCA1	0.19	0.43	0.41
PE/PPE	2G38	PPE	PE	0.41	0.57	0.68

Supplementary Table 3. Performance of the 3 protein-protein docking packages DOT2, FroDOCK and ZDOCK for a benchmark set including 4 protein-protein complexes. Listed in the last 3 columns are the RMS deviations between the Calpha-atoms of X-ray or NMR structure and the predicted complex with the most favorable docking score.

		DOT2 (the lower, the better) kcal/mol	FroDOCK (the higher, the better) arbitrary unit	ZDOCK (the higher the better) arbitrary unit
STIM1		-19.942 (-19.942)	1030.933 (1030.933)	856.502 (856.502)
STIM2.2		-16.733 (-17.242)	912.942 (973.020)	846.201 (990.539)
STIM2.1	Model 1	N/A	N/A	N/A
	Model 2	-13.570 (-13.660)	1160.013 (1215.466)	1072.326 (1111.665)
	Model 3	-13.288 (-15.519)	1032.272 (1185.004)	N/A
	Model 4	-14.105 (-14.693)	N/A	870.845 (1092.589)

Supplementary Table 4. Best scores of conformations or ORAI C-terminal helix docked against STIM motif 1 out of top 30 conformations

Supplementary Methods

Homology modelling and protein-protein docking analyses

The CAD domain (CRAC activation domain) was defined as the segment containing amino acids 342-448 of human STIM1. By definition, the CAD domain covers the entire region of SOAR (STIM1 Orai1-activating region, amino acids 344-442). The X-ray crystallographic structure of the human STIM1 CAD/SOAR dimer was determined by [1] and was deposited in the Protein Data Bank as 3TEQ.pdb. To build a 3D model of the STIM2.2 CAD domain that has 78 % sequence identity with STIM1, we applied homology modeling. The reliability of the homology modeling method is based on the pioneering work by Sander and Schneider [2] who showed that two protein sequences that can be aligned over a stretch of 80 residues or more and show a sequence identity higher than 25% are extremely likely to share similar 3D structures. Thus, we adopted 3TEQ as the structural template [3] and used the homology modeling software MODELLER 9.13 [4]. Due to the way of construction, the STIM2.2 model necessarily matches very closely the STIM1 crystal structure.

Modeling of the new splice variant STIM2.1 carrying the 8-residue insertion "VAASYLIQ" is much more complicated than for STIM2.2. As a word of caution, we point out that the exact conformation of this insertion and its precise positioning in STIM2.1 cannot be predicted with ultimate confidence with current computational resources and algorithms. To propose the most likely secondary structure of this 8-residue insertion, we performed a similarity search against the full PDB database. The top hit structure is a 7-residue peptide VAAYLIQ of protein tyrosine phosphatase (PDB ID: 3RGQ) that adopts an α-helical conformation but lacks the central serine residue. As an independent source of information, we submitted the entire sequence of human STIM2.1 to the established protein structure prediction servers PredictProtein [5], PHD [6] and PSIPRED [7]. All servers predicted for this region an α-helical conformation as most likely conformation. The confidence scores for the 8 positions are "66666766" (PredictProtein), "89999999" (PHD) and "87767899" (PSIPRED). In addition, we performed an 100 ns long self-guided Langevin dynamics simulation [8] for a 16 amino acid peptide with the sequence "AKDEVAASYLIQAEKI". This sequence is that of the 8-residue insertion flanked by 4-residue extensions at both N-terminal and C-terminal ends adopted from the STIM2.1 sequence. As initial conformation of the peptide, we used an unfolded and stretched conformation. During the 100 ns long simulation, the peptide folded and stayed a substantial time in alpha-helical conformation as shown in the figure below (Supplementary Figure 8). All these approaches suggest the 8-residue insertion to be helical. By applying the function of multiple templates (Supplementary Figure 9) of MODELLER to constrain amino acids 384-391 to an α-helical structure of roughly two turns in length (see Figure 7), we generated a homology model of the STIM2.1 CAD domain where the 8-residue insertion of STIM2.1 simply leads to an extension of the respective helix (Sα1). We termed this STIM2.1 conformation as model 1. Note that the extension of the helix by 8 residues shifts and rotates the residues following this insertion with respect to the STIM1 crystal structure.

To account for possible ambiguities in that the available secondary structure predictions cannot guarantee the 8-residue insertion to adopt a helical structure, we considered three further possibilities for the STIM2.1 conformation where we assumed the 8-residue insertion to be only partly helical, or to completely adopt a loop conformation. For model 2, we built the model exactly according to the sequence alignment between the STIM1 and STIM2.1 CAD domains. In this case, the inserted 8 residues of

STIM2.1 were modeled as a flexible loop. In model 3, the first half segment “VAAS” was constrained to be in helical structure and the other half “YLIQ” was modeled as loop. For model 4, “VAAS” was modeled as loop whereas “YLIQ” was constrained to be helical (Supplementary Figure 4).

All homology models of STIM2 and the X-ray structure of STIM1 were evaluated by the Swiss-Model Structure Assessment server (http://swissmodel.expasy.org/workspace/) after an initial energy minimization. This server evaluates the quality of protein structures by a Qmean6 score [9]. According to Supplementary Table 2, all models have Qmean6 score higher than 0.5. Surprisingly, the STIM2 model has even a slightly better score than the STIM1 X-ray crystal structure. We also performed structural evaluations by Procheck [10]. All our models have 99% of the residues in either the core region or in the allowed region of the Ramachandran plot. Note that STIM2.1 model 1 has the highest percentage of residues in the core region.

Protein-protein docking calculations were then performed with the aim of discriminating between the interaction types of STIM/Orai1 complexes. Docking was performed with three established docking packages DOT2 [11], FRODOCK [12] and ZDOCK [13]. The three programs treat biomolecules (proteins and nucleic acids) as rigid bodies and generate complex conformations by considering shape complementary (or vdW interaction) and electrostatic interactions between the biomolecules as well as desolvation of the binding interfaces. Before applying protein-protein docking in a predictive scenario to STIM/Orai1interactions, we performed a "redocking" benchmark for reproducing bound conformations of four protein complexes formed by SMRT/GPS2 [14], MST1/RASSF5 SARAH domain [15], BRCA1/BARD1 [16] and PE/PPE [17]. The binding modes of these four water-soluble protein complexes are based on helix-helix interactions what is closely related to the case of STIM/ORAI1. Supplementary Table 3 shows the RMSD deviation of the docking models with the most favorable docking scores obtained with the three docking packages (DOT2, FRODOCK and ZDOCK) from the correct X-ray or NMR conformations. All the best scored conformations show small $RMSD_{CA}$ values below 0.8 Å from the native structures.

Before docking of the STIM/Orai systems, the STIM1 CAD crystal structure, the STIM2.2 and STIM2.1 model 1 to model 4 homology models and the NMR structure of the C-terminal helix of Orai1 (amino acids 272-291, PDB ID: 2MAK) were first energy minimized by the Amber9 package [18] to relax some steric clashes between side chain atoms. For this, an implicit solvent model (generalized Born model) was used and all backbone atoms were harmonically restrained to the starting geometry during the energy minimization using a force constant of 8.0 kcal/Å-mol. During the protein-protein docking calculations with DOT2, FRODOCK and ZDOCK, the Orai1 C-terminal helix was set as mobile object whereas the STIM CAD monomer structures were set as stationary objects.

As mentioned in the main text, the ORAI C-terminal helix docked against the STIM proteins in different regions that we termed motif 1, motif 2, and motif 3. We point out that docking motif 1 identified for the Orai1:STIM1 interaction appears most plausible. We do not expect that the Orai1-helix will bind to STIM2.2/STIM2.1 using motifs 2 or 3. Generally, one expects that homologous protein pairs bind in similar orientations [19]. Supplementary Table 4 compares the docking scores for binding to motif 1 compared to the best-scoring motif for each case. For STIM1, all 3 docking packages predict motif 1 as the most favorable docking orientation. For STIM2.2, the DOT2 program assigns a slightly weaker binding affinity for motif 1 than the most favorable orientation. This suggests that in the case of STIM2, binding to motif 1 is reduced but still feasible. This would in fact match the experimental finding that

STIM2.2 can still activate Orai1, but with a reduced efficiency. DOT2 further predicts that in the case of STIM2.1, docking to motif 1 (best predicted affinity is -14.105 kcal/mol) is 1.5 kcal/mol more unfavorable than the best docking pose (-15.519 kcal/mol). Also, all docking scores for STIM2.1 are all clearly lower than for STIM1 and STIM2.2. The results obtained with the two other software packages do not allow us to conclude on the efficiency of Orai1-stimulation by STIM2.2 vs. STIM2.1. However, we point that the DOT2 package applies - in our view - the most detailed model for electrostatic interactions. Thus, we believe that it is most suitable among these 3 packages to treat electrostatically dominated interactions as is the case here.

Supplementary References

1 Yang, X., Jin, H., Cai, X., Li, S. & Shen, Y. Structural and mechanistic insights into the activation of Stromal interaction molecule 1 (STIM1). *Proc Natl Acad Sci U S A* **109**, 5657-5662, (2012).

2 Sander, C. & Schneider, R. Database of homology-derived protein structures and the structural meaning of sequence alignment. *Proteins* **9**, 56-68 (1991).

3 Wang, X. *et al.* Distinct Orai-coupling domains in STIM1 and STIM2 define the Orai-activating site. *Nat Commun* **5**, 3183 (2014).

4 Eswar, N. *et al.* Comparative protein structure modeling using Modeller. *Curr Protoc Bioinformatics* **Chapter 5**, Unit 5 6 (2006).

5 Yachdav, G. *et al.* PredictProtein--an open resource for online prediction of protein structural and functional features. *Nucleic Acids Res* **42**, W337-343 (2014).

6 Rost, B. & Sander, C. Prediction of protein secondary structure at better than 70% accuracy. *J Mol Biol* **232**, 584-599 (1993).

7 Buchan, D. W., Minneci, F., Nugent, T. C., Bryson, K. & Jones, D. T. Scalable web services for the PSIPRED Protein Analysis Workbench. *Nucleic Acids Res* **41**, W349-357 (2013).

8 Wu, X. W. & Brooks, B. R. Self-guided Langevin dynamics simulation method. *Chem Phys Lett* **381**, 512-518 (2003).

9 Benkert, P., Schwede, T. & Tosatto, S. C. QMEANclust: estimation of protein model quality by combining a composite scoring function with structural density information. *BMC Struct Biol* **9**, 35 (2009).

10 Laskowski, R. A., Macarthur, M. W., Moss, D. S. & Thornton, J. M. Procheck - a Program to Check the Stereochemical Quality of Protein Structures. *J Appl Crystallogr* **26**, 283-291 (1993).

11 Roberts, V. A., Thompson, E. E., Pique, M. E., Perez, M. S. & Ten Eyck, L. F. DOT2: Macromolecular docking with improved biophysical models. *J Comput Chem* **34**, 1743-1758 (2013).

12 Garzon, J. I. *et al.* FRODOCK: a new approach for fast rotational protein-protein docking. *Bioinformatics* **25**, 2544-2551 (2009).

13 Pierce, B. G., Hourai, Y. & Weng, Z. Accelerating protein docking in ZDOCK using an advanced 3D convolution library. *PLoS One* **6**, e24657 (2011).

14 Oberoi, J. *et al.* Structural basis for the assembly of the SMRT/NCoR core transcriptional repression machinery. *Nat Struct Mol Biol* **18**, 177-184 (2011).

15 Hwang, E. *et al.* Structural basis of the heterodimerization of the MST and RASSF SARAH domains in the Hippo signalling pathway. *Acta Crystallogr D Biol Crystallogr* **70**, 1944-1953 (2014).

16 Brzovic, P. S., Rajagopal, P., Hoyt, D. W., King, M. C. & Klevit, R. E. Structure of a BRCA1-BARD1 heterodimeric RING-RING complex. *Nat Struct Biol* **8**, 833-837 (2001).

17 Strong, M. *et al.* Toward the structural genomics of complexes: crystal structure of a PE/PPE protein complex from Mycobacterium tuberculosis. *Proc Natl Acad Sci U S A* **103**, 8060-8065 (2006).

18 Case, D. A. *et al.* The Amber biomolecular simulation programs. *J Comput Chem* **26**, 1668-1688 (2005).

19 Aloy, P., Ceulemans, H., Stark, A. & Russell, R. B. The relationship between sequence and interaction divergence in proteins. *J Mol Biol* **332**, 989-998 (2003).
